# The role of mitochondrial DNA copy number in cardiometabolic disease: a bidirectional two-sample mendelian randomization study

**DOI:** 10.1186/s12933-023-02074-1

**Published:** 2024-01-28

**Authors:** Pei Qin, Tianhang Qin, Lei Liang, Xinying Li, Bin Jiang, Xiaojie Wang, Jianping Ma, Fulan Hu, Ming Zhang, Dongsheng Hu

**Affiliations:** 1 Department of General Practice, The Affiliated Luohu Hospital of Shenzhen University, No. 47, Youti Road, Shenzhen, 518001 Guangdong China; 2 Institute of Software Chinese Academy of Sciences, Beijing, Guangdong China; 3 Department of Gynecology and Obstetrics, Shenzhen Qianhai Shekou Free Trade Zone Hospital, Shenzhen, Guangdong China; 4 School of Public Health, Shantou University, Shantou, Guangdong China; 5 Department of Neurology, Shenzhen Qianhai Shekou Free Trade Zone Hospital, Shenzhen, Guangdong China; 6 School of Public Health, Shenzhen University Health Science Center, Shenzhen, Guangdong China

**Keywords:** Mitochondrial DNA copy number, Cardiometabolic disease, Bidirectional, Two-sample mendelian randomization study

## Abstract

**Background:**

This study used a bidirectional 2-sample Mendelian randomization study to investigate the potential causal links between mtDNA copy number and cardiometabolic disease (obesity, hypertension, hyperlipidaemia, type 2 diabetes [T2DM], coronary artery disease [CAD], stroke, ischemic stroke, and heart failure).

**Methods:**

Genetic associations with mtDNA copy number were obtained from a genome-wide association study (GWAS) summary statistics from the UK biobank (n = 395,718) and cardio-metabolic disease were from largest available GWAS summary statistics. Inverse variance weighting (IVW) was conducted, with weighted median, MR-Egger, and MR-PRESSO as sensitivity analyses. We repeated this in the opposite direction using instruments for cardio-metabolic disease.

**Results:**

Genetically predicted mtDNA copy number was not associated with risk of obesity (P = 0.148), hypertension (P = 0.515), dyslipidemia (P = 0.684), T2DM (P = 0.631), CAD (P = 0.199), stroke (P = 0.314), ischemic stroke (P = 0.633), and heart failure (P = 0.708). Regarding the reverse directions, we only found that genetically predicted dyslipidemia was associated with decreased levels of mtDNA copy number in the IVW analysis (β= − 0.060, 95% CI − 0.044 to − 0.076; P = 2.416e−14) and there was suggestive of evidence for a potential causal association between CAD and mtDNA copy number (β= − 0.021, 95% CI − 0.003 to − 0.039; P = 0.025). Sensitivity and replication analyses showed the stable findings.

**Conclusions:**

Findings of this Mendelian randomization study did not support a causal effect of mtDNA copy number in the development of cardiometabolic disease, but found dyslipidemia and CAD can lead to reduced mtDNA copy number. These findings have implications for mtDNA copy number as a biomarker of dyslipidemia and CAD in clinical practice.

**Supplementary Information:**

The online version contains supplementary material available at 10.1186/s12933-023-02074-1.

## Introduction

Mitochondria play a vital role in the cellular energy metabolism, cellular differentiation, proliferation, reprogramming, and aging. Mitochondrial DNA (mtDNA), mitochondria’s own genome, encodes 2 ribosomal RNAs, 22 transfer RNAs, and 13 polypeptides of the respiratory chain [1]. The mitochondrion contains multiple copies of mtDNA, and cells contain up to 7000 mitochondria per cell [2]. mtDNA copy number, reflecting the ratio of mitochondrial to nuclear DNA copies, is considered a surrogate for the number of mitochondria [3] and mitochondrial dysfunction and can indirectly reflect mtDNA damage [4]. Cardiometabolic disease is characterized by mitochondrial dysfunction, oxidative stress, impaired oxidative phosphorylation, and inflammation [5]. Considering the inexpensiveness and accessibility of the mitochondrial DNA copy number, it has recently garnered interest to be used as a biomarker of chronic diseases.

Cardiometabolic disease is a global health issue with an increasing disease burden and leading causes of global death and disability [6]. Some previous cross-sectional, case–control, and prospective epidemiological studies as well as meta-analyses have reported an increased risk of cardiovascular disease associated with decreased levels of mtDNA copy number [7–10]. mtDNA copy number was also reported to be associated with increased risk of hypertension [11], obesity [12], and diabetes [13, 14], in the observational studies. A large cross-sectional study including 408,361 participants of multiple ancestries in eight US cohorts from the Trans-Omics for Precision Medicine and UK Biobank reported the significant negative associations of whole blood mtDNA copy number with several cardiometabolic disease traits including obesity, hypertension, diabetes, and hyperlipidemia [15]. However, so far, it remains unclear whether there is a causal relationship between mtDNA copy number and these diseases. Moreover, it remains to be addressed whether there is reverse causality that cardiometabolic diseases affect mtDNA copy number.

Mendelian randomization (MR) is a method to use genetic variants randomly allocated during conception as instrumental variables for exposure to estimate the causal effect of an exposure on an outcome in observational data, which is a powerful approach that can avoid potential bias by confounders and reverse causation [16]. The causal relationship between mtDNA copy number and risk of diabetes, stroke, and stroke prognosis has been previously studied via MR design [17, 18]. However, the association of mtDNA copy number and other cardiometabolic disease (e.g., obesity, hypertension, hyperlipidaemia, coronary artery disease [CAD], and heart failure) has not yet been investigated using the MR design. Furthermore, the reverse relationship using MR design is also lacking except for diabetes and CAD.

Here, in the present study, we performed a bidirectional 2-sample MR analysis to assess the bidirectional causality between mtDNA copy number and risk of cardiometabolic outcomes, including obesity, hypertension, hyperlipidaemia, type 2 diabetes mellitus (T2DM), CAD, stroke, ischemic stroke (IS), and heart failure.

## Methods

### Overall study design

We performed bidirectional 2-sample MR analyses based on the latest summary statistics of genome-wide association studies (GWASs) to investigate the associations between mtDNA copy number and cardiometabolic disease as well as to test whether cardiometabolic disease causes a change in the mtDNA copy number. MR uses single nucleotide polymorphisms (SNPs) as instrumental variables to test the causal effect of risk factors with an outcome, which will not be influenced by reverse causation bias and any confounding factors as SNPs are randomly allocated at meiosis based on Mendel’s laws. Three assumptions should be met for MR analysis: (1) the SNPs are associated with the exposure; (2) the SNPs are independent from confounders of the exposure-outcome relation (the independence assumption); and (3) the SNPs affect the outcome only through the exposure [19]. The study design of the present MR analysis consisted of 8 cardiometabolic outcomes, including obesity, essential hypertension, hyperlipidemia, T2DM, CAD, stroke, IS, and heart failure. All the summary data used in the study are publicly available, and the detailed information is shown in Table 1.

**Table 1:** Characteristics of the used genome-wide association study in the study

Phenotypes	Study/consortium	Cases/ controls	Adjusted variables	PubMed ID
Mitochondrial DNA copy number	UK Biobank	395,718	Age, age2, sex, chip type, 20 genetic principal components, and blood cell counts (white blood cell, platelet, and neutrophil counts)	35023831
Mitochondrial DNA copy number in the replication analysis	CHARGE and UK Biobank	465,809	Age, sex, principal components, DNA collection site, family structure and cell composition	34859289
Obesity	FinnGen consortium	18,330/324,070	Age, sex, genotyping batch, 10 principal components	–
Hypertension	FinnGen consortium	98,683/243,756	Age, sex, genotyping batch, 10 principal components	–
Dyslipidemia	FinnGen consortium	22,460/296,380	Age, sex, genotyping batch, 10 principal components	–
T2DM	DIAGRAM	74,124/824,006	BMI	30297,969
CAD	CARDIOGRAMPLUSC4D	60,801/123,504	Sex, age, and generation (Original or Offspring Cohort)	26343387
Stroke	MEGASTROKE	40,585/406,111	Age, sex	29531354
Ischemic stroke	MEGASTROKE	34,217/406,111	Age, sex	29531354
Heart failure	HERMES	47,309/930,014	Age, sex, and principal components	31919418

*BMI* body mass index, *CAD* coronary artery disease, *CARDIoGRAMplusC44D* Coronary Artery Disease Genome wide Replication and Meta-analysis (CARDIoGRAM) plus the Coronary Artery Disease (C4D) Genetics consortium, *CHARGE* Heart and Aging Research in Genomic Epidemiology, *DIAGRAM Consortium* DIAbetes Genetics Replication and Meta-analysis Consortium, *HERMES* the Heart Failure Molecular Epidemiology for Therapeutic Targets Consortium, *T2DM* type 2 diabetes mellitus

This study is reported in accordance with the Strengthening the Reporting of Observational Studies in Epidemiology Using Mendelian Randomization (STROBE-MR) reporting guideline [20]. All studies included in the GWASs and consortia that were used in the present study had been approved by a relevant review board and involved participants had given informed consent.

### Data sources

#### mtDNA copy number

GWAS summary statistics for mtDNA copy number were derived from 395,718 UK Biobank participants of various ancestries (mostly European). mtDNA copy number estimates were ascertained using the AutoMitoC pipeline developed by Chong et al. [21], which represents the most comprehensive genetic assessment published to date than previous investigations for mtDNA copy number [22, 23]. The GWAS adjusted for age, age^2^, sex, chip type, 20 genetic principal components, and blood cell counts (white blood cell, platelet, and neutrophil counts). In the validation study, GWAS summary statistics associated with mtDNA copy number in 465,809 White individuals from the Cohorts for Heart and Aging Research in Genomic Epidemiology (CHARGE) consortium and the UK Biobank (UKB) by Longchamps et al. 2022 was used [22].

#### Cardiometabolic diseases

We used summary statistics from the largest available published GWAS of cardiometabolic disease of interest. Relevant information on the GWAS summary statistics is presented in Table 1. GWAS Summary statistics for obesity (18,330 cases and 324,070 controls), hypertension (98,683 cases and 243,756 controls), and dyslipidaemia (22,460 cases and 296,380 controls) were derived the FinnGen consortium released in 2021 (https://r8.finngen.fi/). In terms of T2DM, we used the genome-wide data from a meta-analysis conducted by the DIAbetes Genetics Replication And Meta-analysis (DIAGRAM) consortium, which included 74,124 T2DM cases and 824,006 controls from 32 European-ancestry studies [24]. We extracted the GWAS summary statistics of CAD from the Coronary Artery Disease Genome-wide Replication and Meta-analysis Plus the Coronary Artery Disease Genetics (CARDIoGRAMplusC4D) (http://www.cardiogramplusc4d.org/data-downloads/), which is a meta-analysis of 48 studies with 60,801 CAD cases and 123,504 controls of European (77%), South Asian (13%), East Asian (6%), and Hispanic and African Americans ancestry (4%) [25]. The diagnosis of CAD included myocardial infarction, acute coronary syndrome, chronic stable angina, or coronary artery stenosis of at least 50%. For the summary statistics of stroke and IS, we used a recent large-scale meta-analysis of GWAS (MEGASTROKE) confined to European populations which included 446,696 individuals of European ancestry (406,111 noncases and 40,585 cases of any stroke); the number of IS cases were 34,217 overall [26]. Summary statistics for heart failure were obtained from 25 meta-analysis of 26 European-ancestry GWASs with 47,309 heart failure cases and 930,014 controls by Shah et al. [27].

### Definition of cardiometabolic diseases

Obesity (E66), hypertension (I10 to I13, I15, I674), and dyslipidaemia (E780) were defined according to the ICD-10 (https://www.finngen.fi/en/researchers/clinical-endpoints). T2DM in the selected GWAS was defined by diagnostic fasting glucose, casual glucose, 2 h plasma glucose or HbA1c levels; use of glucose-lowering medication (by Anatomical Therapeutic Chemical code or self-report); or T2DM history from medical records or self-report [24]. CAD was defined by an inclusive CAD diagnosis including myocardial infarction (MI), acute coronary syndrome, chronic stable angina, or coronary stenosis > 50% [25]. Stroke was defined by rapidly developing signs of focal (or global) disturbance of cerebral function, lasting ≥ 24 h or leading to death due to vascular origin without apparent other causes according to the World Health Organization. IS was defined based on clinical and imaging criteria [26]. The study-specific stroke and IS ascertainment are described in the published study [26]. HF cases were defined as those with a clinical diagnosis of HF of any actinology with no inclusion criteria based on left ventricular ejection function [27]. Definitions of HF status within each study in the meta-analysis were described in detail in the study [27].

### Instruments selection

Strict selection criteria were used to select qualified instrumental variables. We included all SNPs at the genome-wide significance level (*P* < 5 × 10^−8^) and pruned all SNPs with the stringent pairwise linkage disequilibrum (LD) *r*^2^ < 0.001 and clumping distance > 10,000 kb. We then applied the PhenoScanner V2 [28] (http://www.phenoscanner.medschl.cam.ac.uk/phenoscanner, accessed on 31 May 2023) to evaluate whether the genetic instruments were associated with other phenotypes. Steiger filtering was used to remove SNPs that were correlated with outcomes stronger than exposures [29]. Instrumental strength for the SNP-exposure association was measured by averaging SNP-specific F-statistics which was calculated by the square of the beta divided by the variance for the SNP–exposure association. A weak instrumental variable was defined as an F-statistic less than 10, and all weak instrumental variables were excluded [30].

### Statistical analyses

A generalized inverse variance-weighted (IVW) approach MR (IVW MR) under a multiplicative random-effects model was applied as the principal analysis. Cochrane’s Q was used to assess the heterogeneity of estimates of SNPs, and a *P* < 0.05 was considered significant in the test for heterogeneity. The weighted median [31], MR-Egger [32], Mendelian Randomization Pleiotropy RESidual Sum and Outlier (MR-PRESSO) [33] were used in the sensitivity analyses to examine the consistency of associations and detect and correct for horizontal pleiotropy. The weighted median method was used to check invalid instrument bias. This method provides a consistent estimate if over 50% of the weight in the meta-analysis has been derived from valid SNPs. MR-Egger was used to identify potential directional pleiotropy. The P-value for intercept in MR-Egger analysis was used to assess the horizontal pleiotropy (*P* < 0.05). MR-PRESSO analysis was conducted to discern and correct for the potential horizontal pleiotropic outliers. The leave-one-out method was implemented by sequentially excluding each SNP to determine whether the estimates were driven by any single SNP.

Results were reported as odds ratio (OR) with corresponding 95% CIs or β (SE). The statistical analyses were performed with R packages MendelianRandomization, MRPRESSO, TwoSampleMR package using the statistical software R (version 4.1.2; R Foundation for Statistical Computing). To account for the high number of comparisons being made between mtDNA copy number and cardiometabolic disease (and vice versa) (n = 16 tests), we used a Bonferroni adjustment to all *P*-value thresholds, with the threshold of statistical significance of 0.003 (0.05/16). *P* ≤ 0.05 but not reaching the Bonferroni corrected significance threshold was suggestive of evidence for a potential causal association.

## Results

**Fig. 1:**
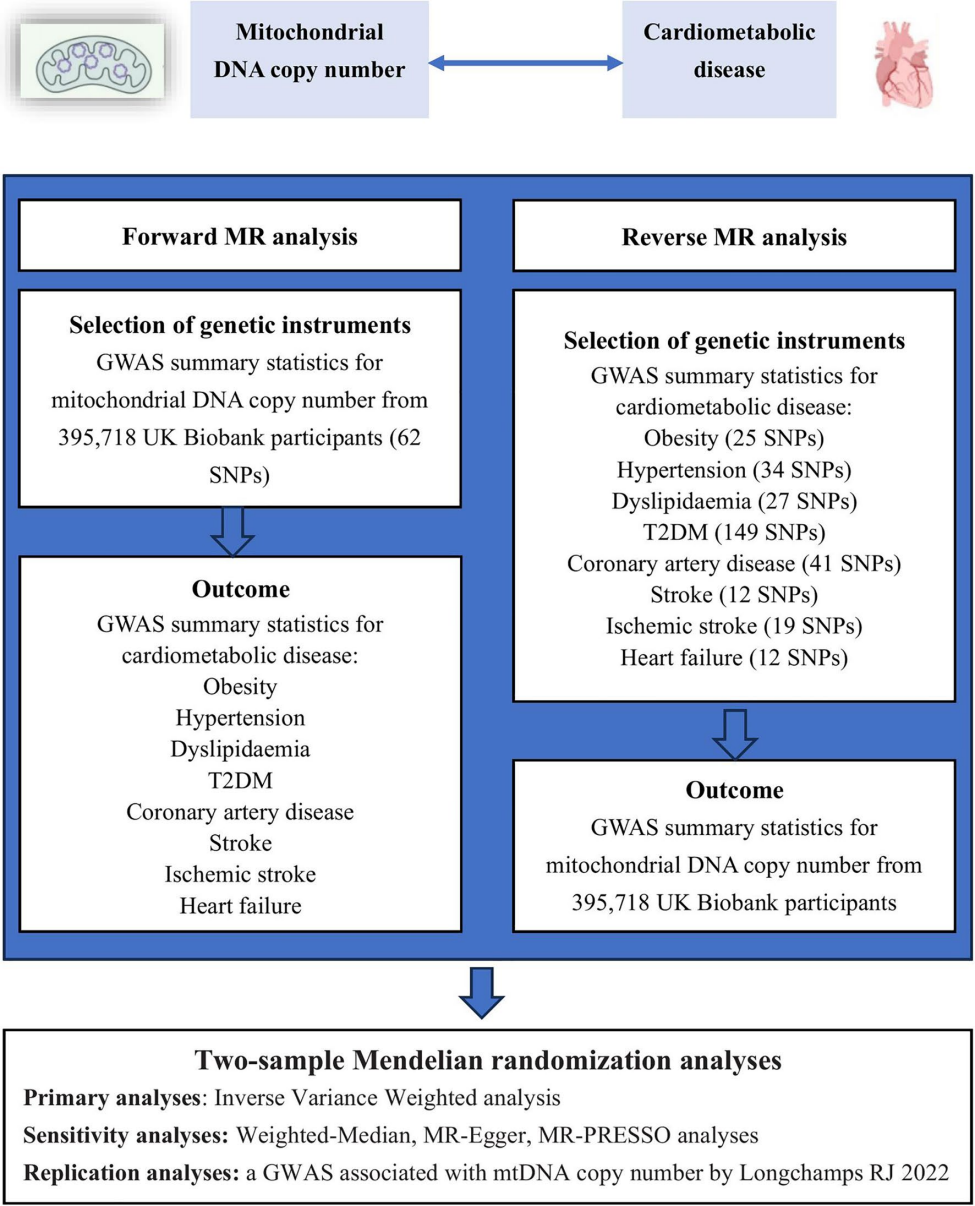
Overview of study design of the bidirectional Mendelian randomization framework used to investigate the causal effect of mitochondrial DNA copy number on cardiometabolic disease. We performed a total of 16 MR analyses to investigate the bidirectional association between mitochondrial DNA copy number on cardiometabolic disease including obesity, hypertension, dyslipidemia, type 2 diabetes mellitus, coronary artery disease, stroke, ischaemic stroke, and heart failure. All genetic instruments were single nucleotide polymorphisms (SNPs). T2DM, type 2 diabetes mellitus

The flowchart of the bi-directional MR analysis is shown in Fig. 1.

### Association of genetically predicted mtDNA copy number with cardiometabolic disease

A total of 6694 SNPs associated with mtDNA copy number at genome-wide significance (*P* < 5 × 10^−8^) were obtained. After further dropping 6628 SNPs due to linkage disequilibrium reference panel or high linkage disequilibrium (*r*^2^ > 0.001), 66 remained in the main analysis. The F statistics for the associations of genetic instruments with mtDNA copy number was 93.31 (range from 29.54 to 473.58). After removing rs4841132 associated with fasting blood glucose and insulin, rs7896518 associated with BMI, rs6511720 associated with low-density lipoprotein and CAD, rs8176645 associated with lipids profile, 62 SNPs for mtDNA copy number were contained in the association between mtDNA copy number and cardiometabolic disease (Additional file 1: Table S1). The SNPs explained 29.8% of the variance in the mtDNA copy number.

**Fig. 2:**
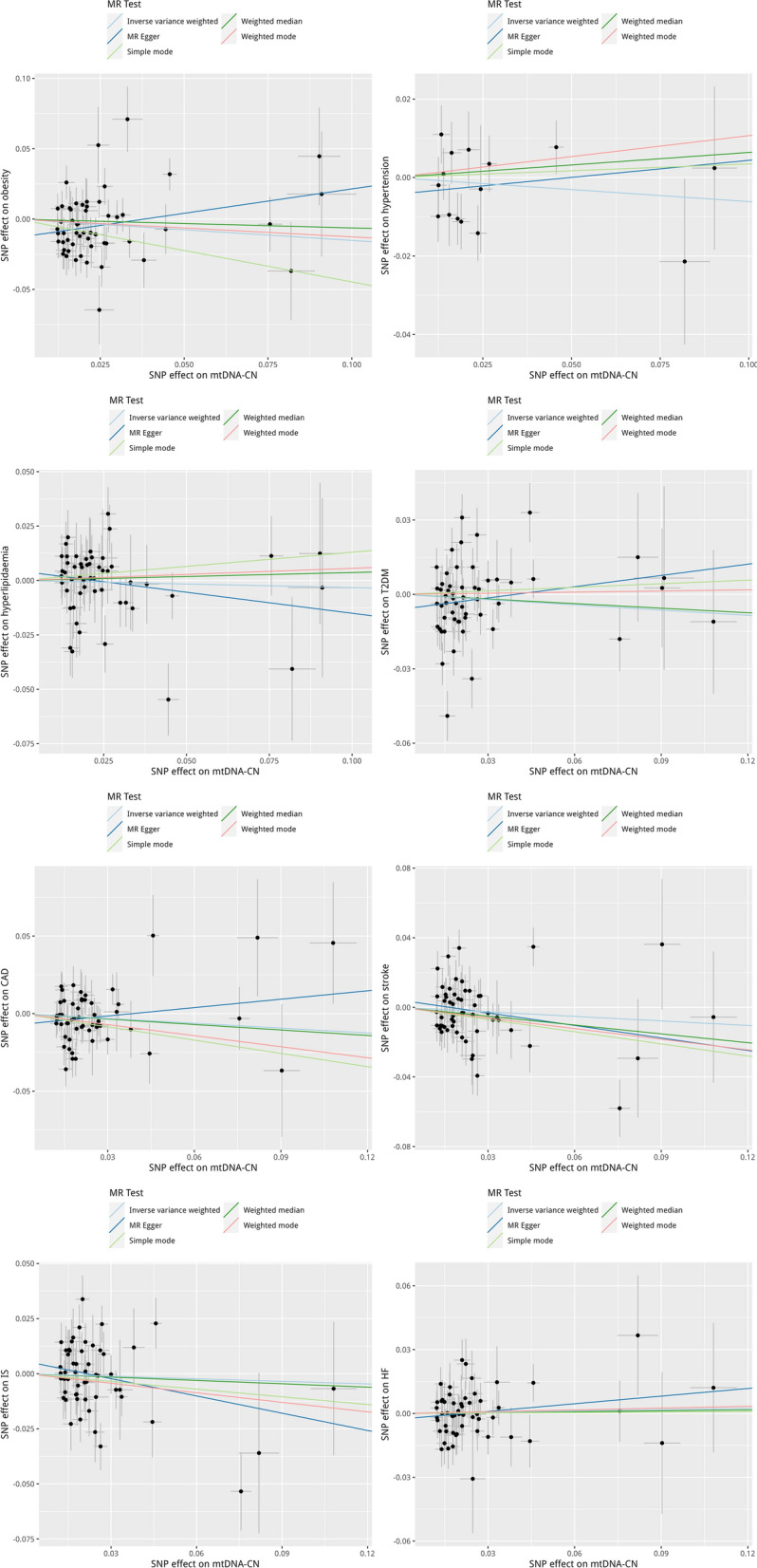
The forward MR analyses: Scatter plot of the association between mtDNA copy number and cardiometabolic disease. **A** Obesity, **B** hypertension, **C** dyslipidemia, **D** T2DM, **E** CAD, **F** stroke, **G** ischemic stroke, **H** heart failure. Lines in black, red, green, and blue represent IVW, MR-Egger, weighted median, and weight mode methods. *CAD* coronary artery disease, *HF* heart failure, *IVW* inverse variance weighting, *IS* ischemic stroke, *mtDNA-CN* mtDNA copy number, *SNPs* single nucleotide polymorphisms, *T2DM* type 2 diabetes mellitus

Genetically predicted mtDNA copy number was not associated with obesity (OR = 0.859, 95% CI 0.699–1.055; *P* = 0.148), hypertension (OR = 0.941, 95% CI 0.782–1.131; *P* = 0.515), dyslipidemia (OR = 0.968, 95% CI 0.828–1.132; *P* = 0.684), T2DM (OR = 0.962; 95% CI 0.822–1.127; *P* = 0.631), CAD (OR = 0.901; 95% CI 0.768–1.056; *P* = 0.199), stroke (OR = 0.917, 95% CI 0.775–1.086; *P* = 0.314), ischemic stroke (OR = 0.962, 95% CI 0.822–1.127; *P* = 0.631), and heart failure (OR = 1.021; 95% CI 0.917–1.135; *P* = 0.708) using the primary IVW analysis. Scatter plot for the forward analyses and the plots of “leave-one-out” analyses for each SNP-cardiometabolic disease association are summarized in the Fig. 2 and Additional file 1: Fig. S1, respectively.

Sensitivity analyses using a weighted median, MR-Egger, and MR-PRESSO showed similar null findings, with exception that the causal association of mtDNA copy number with obesity approached statistical significance in the MR-PRESSO (OR = 0.790, 95% CI 0.644–0.969; *P* = 0.028) after removing 1 outlier SNP. There was no indication of possible horizontal pleiotropy from the MR-Egger intercept for all outcomes (Table 2). There was statistical evidence of pleiotropy for obesity (*P* < 0.001), T2DM (*P* = 0.001), CAD (*P* < 0.001), stroke (*P* < 0.001), ischemic stroke (*P* = 0.001), and heart failure (p = 0.001), and no indication of pleiotropy for hypertension and dyslipidemia, with an I^2^ ranging from 22.86 to 69.09% (Table 2).

**Table 2:** Mendelian randomization estimates between genetically predicted mtDNA copy number and the risk of cardiometabolic disease

Exposure	Outcome	No. of SNPs	Methods	OR^a^	Lower 95% CI	Upper 95% CI	P	MR-Egger intercept (P value)	Cochran’s Q test (I^2^)	P	Outliers from MR-PRESSO
mtDNA-CN	Obesity	54	IVW	0.859	0.699	1.055	0.148	0.018	104.092 (69.09%)	< 0.001	rs72660908
			WM	0.939	0.736	1.198	0.613				
			MR-Egger	1.414	0.906	2.206	0.133				
			MRPRESSO	0.790	0.644	0.969	0.028				
mtDNA-CN	Hypertension	15	IVW	0.941	0.782	1.131	0.515	0.357	18.223 (23.17%)	0.197	NA
			WM	1.066	0.851	1.336	0.578				
			MR-Egger	1.091	0.764	1.557	0.640				
			MRPRESSO	0.941	0.756	1.125	0.525				
mtDNA-CN	Dyslipidemia	54	IVW	0.968	0.828	1.132	0.684	0.324	68.710 (22.86%)	0.072	NA
			WM	1.037	0.845	1.273	0.726				
			MR-Egger	0.824	0.579	1.174	0.289				
			MRPRESSO	0.968	0.812	1.124	0.686				
mtDNA-CN	T2DM	55	IVW	0.962	0.822	1.127	0.631	0.209	72.880 (25.90%)	0.044	NA
			WM	0.950	0.782	1.154	0.608				
			MR-Egger	0.770	0.528	1.123	0.180				
			MRPRESSO	0.928	0.773	1.083	0.351				
mtDNA-CN	CAD	57	IVW	0.901	0.768	1.056	0.199	0.114	114.772 (53.82%)	< 0.001	NA
			WM	0.889	0.722	1.095	0.269				
			MR-Egger	1.198	0.818	1.754	0.357				
			MRPRESSO	0.901	0.742	1.060	0.245				
mtDNA-CN	Stroke	55	IVW	0.917	0.775	1.086	0.314	0.390	95.959 (43.7%)	< 0.001	rs1760940, rs2263663
			WM	0.844	0.690	1.032	0.099				
			MR-Egger	0.786	0.534	1.158	0.229				
			MRPRESSO	0.920	0.767	1.074	0.296				
mtDNA-CN	Ischemic stroke	54	IVW	0.962	0.822	1.127	0.631	0.209	89.620 (40.90%)	0.001	NA
			WM	0.950	0.778	1.162	0.620				
			MR-Egger	0.770	0.528	1.123	0.180				
			MRPRESSO	0.962	0.804	1.120	0.633				
mtDNA-CN	Heart failure	57	IVW	1.021	0.917	1.135	0.708	0.378	89.620 (40.86%)	0.001	NA
			WM	1.014	0.861	1.194	0.868				
			MR-Egger	1.127	0.884	1.437	0.340				
			MRPRESSO	1.021	0.916	1.125	0.705				

*CAD* coronary artery disease, *T2DM* type 2 diabetes mellitus, *PRESSO* Pleiotropy Residual Sum and Outlier

^a^Odds ratios for associations between genetically predicted mtDNA copy number and cardiometabolic disease. The ORs represent the odds ratios per 1-standardized unit (in SD unit) increase in the mtDNA copy number. The random-effects inverse variance-weighted method was used as the primary approach, while other methods including MR-Egger, weighted median-based, MR-PRESSO were used as sensitivity analyses. The MR-PRESSO global test and MR-Egger regression were adopted to detect the pleiotropic effects. The MR-Egger regression method was to detect the effect of genetic instruments on the exposure which is plotted against its effect on the outcome, and an intercept distinct can be used to identify whether there are pleiotropic effects (MR-Egger regression test: p < 0.01). MR-PRESSO was used to calculate the outlier-corrected MR estimates if the horizontal pleiotropy was present (MR-PRESSO global test: p < 0.01). The q values derived from the Cochran’s Q statistics were used to reflect heterogeneity between the SNP-specific estimates, and the weighted median-based results should be highlighted if significant heterogeneity was observed

Detailed information on the SNPs of Longchamps’s GWAS is presented in Additional file 1: Table S3. The non-significant associations were confirmed by the validation sample based on 133 SNPs for mtDNA copy number from the summary statistics (Additional file 1: Table S4), except that the association between mtDNA copy number and CAD (OR = 0.842, 95% CI 0.713–0.994, *P* = 0.042) reached at a nominal *P* value (*P* < 0.05) but not at Bonferroni-corrected significance (*P* < 0.003) in the IVW analysis. Scatter plot for the forward replication analyses and the plots of “leave-one-out” analyses for each SNP-cardiometabolic disease association are summarized in the Additional file 1: Fig S3 and S4, respectively.

**Fig. 3:**
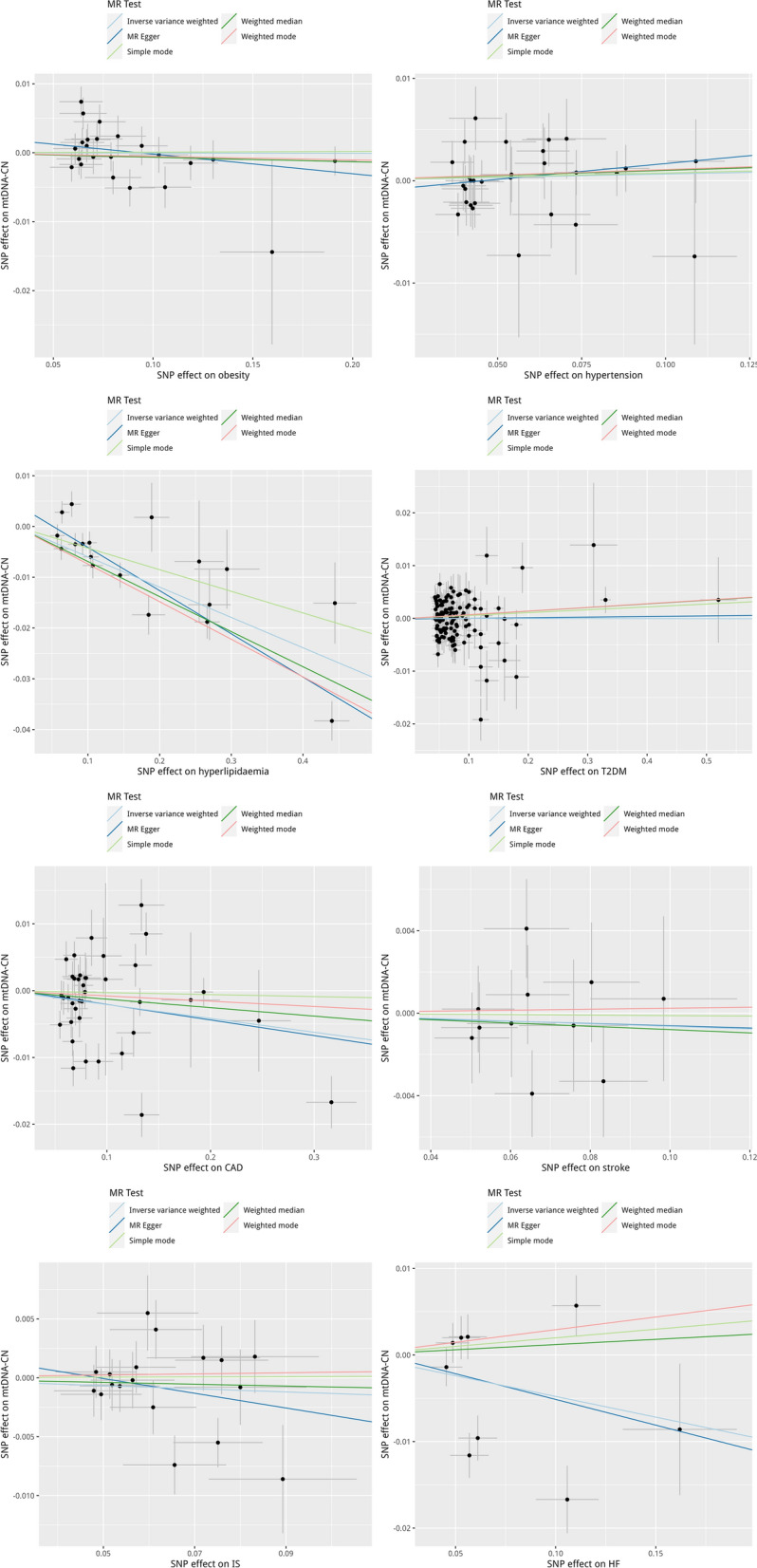
The reverse MR analyses: Scatter plot of the association between cardiometabolic disease and mtDNA copy number. **A** Obesity, **B** hypertension, **C** dyslipidemia, **D** T2DM, **E** CAD, **F** stroke, **G** ischemic stroke, **H** heart failure. Lines in black, red, green, and blue represent IVW, MR-Egger, weighted median, and weight mode methods. *CAD* coronary artery disease, *HF* heart failure, *IVW* inverse variance weighting, *IS* ischemic stroke, *mtDNA-CN* mtDNA copy number, *SNPs* single nucleotide polymorphisms, *T2DM* type 2 diabetes mellitus

### Association of genetically predicted cardiometabolic disease with mtDNA copy number

In the reverse MR analyses of the association between cardiometabolic disease and mtDNA copy number, we included 25 SNPs for obesity, 34 SNPs for hypertension, 27 SNPs for dyslipidemia, 149 SNPs for T2DM, 41 SNPs for CAD, 12 SNPs for stroke, 19 SNPs for ischemic stroke, and 12 SNPs for HF. Variance explained by the SNPs for cardiometabolic disease ranged from 2.5 to 46.6%. The genetic variants used as instrumental variables for the cardiometabolic disease in the reverse MR analyses are presented in Additional file 1: Table S2.

There was no strong evidence for associations of obesity, hypertension, T2DM, stroke, IS, heart failure, or ischemic stroke with mtDNA copy number, whereas the causal association of dyslipidemia (β = − 0.060, 95% CI − 0.044 to − 0.076; *P* = 2.416e−14) and CAD (beta = − 0.021; 95% CI = − 0.003 to − 0.039; *P* = 0.025) with mtDNA copy number showed suggestive statistical significance (Table 3). No horizontal pleiotropy was observed for all cardiometabolic outcomes. The results in sensitivity analyses showed similar findings (Table 3). Scatter plot for the reverse analyses and the plots of “leave-one-out” analyses for each SNP of cardiometabolic disease association on mtDNA copy number were summarized in Fig. 3 and Additional file 1: Fig. S2. The replication analyses by using Longchamps’s GWAS for the reverse association were not performed, due to not enough number of harmonized data.

**Table 3:** Reverse mendelian randomization estimates on associations of genetically predicted cardiometabolic disease and mtDNA copy number

Exposure	Outcome	No. of SNPs	Methods	β	SE	Lower 95% CI	Upper 95% CI	P	MR-Egger intercept (P value)	Cochran’s Q test (I^2^)	P	Outliers from MR-PRESSO
Obesity	mtDNA-CN	25	IVW	− 0.001	0.007	0.013	− 0.015	0.953	0.121	36.200 (36.5%)	0.039	rs7187776
			WM	− 0.007	0.008	0.009	− 0.023	0.432				
			MR-Egger	− 0.029	0.019	0.008	− 0.066	0.142				
			MRPRESSO	− 0.004	0.006	0.008	− 0.016	0.557				
Hypertension	mtDNA-CN	34	IVW	0.007	0.009	0.025	− 0.011	0.457	0.817	25.148 (11.3%)	0.620	NA
			WM	0.010	0.012	0.034	− 0.014	0.417				
			MR-Egger	0.031	0.028	0.086	− 0.024	0.284				
			MRPRESSO	0.007	0.009	0.025	− 0.011	0.457				
Dyslipidemia	mtDNA-CN	26	IVW	− 0.060	0.008	− 0.044	− 0.076	2.416e−14	0.017	46.840 (63.7%)	< 0.001	rs3005923, rs7412
			WM	− 0.069	0.008	− 0.053	− 0.085	5.872e−17				
			MR-Egger	− 0.085	0.012	− 0.061	− 0.109	1.798e−06				
			MRPRESSO	− 0.052	0.007	− 0.038	− 0.066	1.836e−06				
T2DM	mtDNA-CN	149	IVW	− 0.0001	0.004	0.008	− 0.008	0.974	0.852	203.098 (38.5%)	< 0.001	rs10401969
			WM	0.007	0.005	0.017	− 0.003	0.153				
			MR-Egger	0.001	0.008	0.017	− 0.015	0.880				
			MRPRESSO	− 0.002	0.003	0.004	− 0.008	0.766				
CAD	mtDNA-CN	41	IVW	− 0.021	0.009	− 0.003	− 0.039	0.025	0.895	218.390 (78.0%)	< 0.001	rs115654617, rs1412444, rs2519093, rs3918226, rs4420638, rs515135, rs56289821, rs7528419
			WM	− 0.013	0.008	0.003	− 0.029	0.122				
			MR-Egger	− 0.023	0.022	0.020	− 0.066	0.286				
			MRPRESSO	− 0.010	0.007	0.004	− 0.024	0.138				
Stroke	mtDNA-CN	12	IVW	− 0.004	0.006	0.008	− 0.016	0.452	0.988	147. 600 (85.8%)	< 0.001	NA
			WM	− 0.006	0.004	0.002	− 0.014	0.138				
			MR-Egger	0.016	0.026	0.067	− 0.035	0.557				
			MRPRESSO	− 0.004	0.006	0.008	− 0.016	0.452				
Ischemic stroke	mtDNA-CN	19	IVW	− 0.013	0.012	0.011	− 0.037	0.258	0.457	26.093 (34.8%)	0.073	NA
			WM	− 0.008	0.014	0.019	− 0.035	0.591				
			MR-Egger	− 0.063	0.066	0.066	− 0.192	0.355				
Heart failure	mtDNA-CN	12	IVW	− 0.048	0.034	0.019	− 0.115	0.160	0.378	51.956 (3.93%)	0.554	rs17042102, rs55730499, rs600038
			WM	0.012	0.023	0.057	− 0.033	0.608				
			MR-Egger	− 0.059	0.105	0.147	− 0.265	0.588				
			MRPRESSO	0.004	0.019	0.041	− 0.033	0.829				

Odds ratios for associations between genetically predicted cardiometabolic disease and mtDNA copy number. The ORs represent the odds ratios per 1-standardized unit (in SD unit) increase in the mtDNA copy number. The random-effects inverse variance-weighted method was used as the primary approach, while other methods including MR-Egger, weighted median-based, MR-PRESSO were used as sensitivity analyses. The MR-PRESSO global test and MR-Egger regression were adopted to detect the pleiotropic effects. The MR-Egger regression method was to detect the effect of genetic instruments on the exposure which is plotted against its effect on the outcome, and an intercept distinct can be used to identify whether there are pleiotropic effects (MR-Egger regression test: p < 0.01). MR-PRESSO was used to calculate the outlier-corrected MR estimates if the horizontal pleiotropy was present (MR-PRESSO global test: p < 0.01). The q values derived from the Cochran’s Q statistics were used to reflect heterogeneity between the SNP-specific estimates, and the weighted median-based results should be highlighted if significant heterogeneity was observed

*PRESSO* Pleiotropy Residual Sum and Outlier

## Discussion

To our knowledge, this is the first study to use bidirectional two-sample MR to comprehensively investigate the association of genetic predictors determined mtDNA copy number and cardiometabolic disease. The study did not find any causal association between genetic predicted mtDNA copy number and any cardiometabolic disease including obesity, essential hypertension, hyperlipidemia, T2DM, CAD, stroke, IS, and heart failure. A suggestive effect of dyslipidemia and CAD was found on mtDNA copy number; however, there was no evidence supporting causal relationships between other cardiometabolic disease and mtDNA copy number.

Previous observational studies have explored the relationship of mtDNA copy number and obesity [12], hypertension [11], hyperlipidemia [15], T2DM [13, 14], CAD [7–9], stroke [7, 18], and heart failure [10]. Of these studies, very few studies have focused on the associations of mtDNA copy number with obesity, hypertension, hyperlipidemia, CAD, and heart failure, and controversial findings have been shown for T2DM, CAD, and stroke. For T2DM, conflicting results were reported by observational studies, with some studies showing a negative association [17] and others showing a non-significant association [14]. No association was also indicated between mtDNA copy number and CAD [7], although the Atherosclerosis Risk in Communities (ARIC) study involved 15,792 individuals and the Cardiovascular Health Study (CHS) study involved 5201 participants older than 65 years showed significant association for CAD [7]. One cohort study was conducted in the American population, which included a total of 21,870 participants with a mean follow-up of 13.5 years, found that reduced mtDNA copy number significantly increased the risk of stroke (Hazard Ratio = 1.11, 95% CI = 1.06–1.16) [7]. In another cohort study in the Swedish population including 3,062 middle-aged women with mean follow-up of 17 years, mtDNA copy number was not found to be significantly associated with stroke risk (HR = 1.26, 95% CI 0.87–1.84) [8]. As far as we know, the current study comprehensively estimated the bidirectional causal association of mtDNA copy number and a series of cardiometabolic disease using the MR design for the first time except for T2DM and ischemic stroke. A bi-directional MR analysis by Wang et al. [17] found no evidence for causal associations between blood mtDNA-CN and T2DM, and blood mtDNA-CN and BMI in either direction, which was consistent with our findings. Meanwhile, similar to our finding, the study by Leon G. Martens et al. [34] did not find the causal relation between mtDNA abundance and ischemic stroke. Recent studies showed that mtDNA-CN could be a marker of stroke prognosis [18, 35] and MR study also showed the significant association between genetically determined mtDNA-CN and poststroke prognosis [18], which suggests that mtDNA-CN may be a biomarker of stroke prognosis but not the early predictor of stroke development. Furthermore, we first use MR analysis to explore the relation between mtDNA copy number and other cardiometabolic disease including obesity, hypertension, hyperlipidemia, CAD, and heart failure, while our study did not find any associations.

This study is the first to investigate the reverse direction of mtDNA copy number and cardiometabolic disease. It is mechanically reasonable that the presence of cardiometabolic disease involved oxidative stress [36] and inflammation [37], which may further affect the mtDNA copy number [38]. In the present study, we found the significant association of genetically predicted dyslipidemia and CAD with decreased levels of mtDNA copy number; however, significant heterogeneity was found and insignificant association was found in the sensitivity analysis for CAD. The finding suggests the potential causal effect of dyslipidemia on mtDNA copy number. Case-control studies and cross-sectional studies also showed the alteration of mtDNA copy number in patients with hyperlipidemia [15, 39].

Our study had several strengths. Firstly, our study adopted the two-sample MR analyses leveraging SNPs as instrumental variables to assess the causality for the associations between mtDNA copy number and cardiometabolic diseases, which has the advantage of being less vulnerable to residual confounders and reverse causation because of the random allocation of alleles during the formation of the zygote. Secondly, in addition to confirming the association of mtDNA copy number and the risk of cardiometabolic diseases, this is the first systematical MR study to evaluate the effect of cardiometabolic diseases on mtDNA copy number. Finally, we performed MR analyses based on large-scale GWAS datasets, which enabled us to provide a valid appraisal of the causality for the associations between mtDNA copy number and cardiometabolic diseases with a high statistical power. A better understanding of the role of mtDNA copy number in cardiometabolic diseases not only facilitates a clearer perception of the underlying pathophysiology of cardiometabolic diseases, but also helps to capture the potential biomarker.

However, our study also suffered from several limitations. First, our study was mainly based on Europeans, which reduced the generalizability to populations of non-European ancestry. Further studies in different ethnic populations are needed to confirm our findings. Second, the phenotypic variance of mtDNA copy number as explained by the genetic instruments was small, which may lead to the limited statistical power for the estimation of the association. Third, given that sleep pattern is a complex physiological process involving multiple host and environmental factors, sleep phenotypes may influence the risk of cardiometabolic diseases through these factors. Further studies are warranted to investigate the underlying mechanism, although the MR-Egger regression indicated that there was little directional pleiotropy in this MR study. Fourth, although sensitivity analyses did not show significant evidence of heterogeneity, directional pleiotropy, or outlying effects, bias due to pleiotropy or index event bias cannot be avoided.

In conclusion, our study suggests that dyslipidemia and CAD may causally affect mtDNA copy number, but a causal relationship of mtDNA copy number and cardiometabolic disease remains uncertain. More studies are required to better understand the relationship between mtDNA copy number and cardiometabolic diseases.

### Supplementary Information

**Additional file 1: Table S1.** Genetic variants used as instrumental variables for mitochondrial DNA copy number. **Table S2.** Genetic variants used as instrumental variables for the cardiometabolic disease in the reverse MR analyses. **Table S3.** Genetic variants used as instrumental variables for mitochondrial DNA copy number by Longchamps RJ et al. **Table S4.** Replication analyses for the MR analyses on the forward associations of mitochondrial DNA copy number with cardiometabolic diseases using GWAS summary data of mitochondrial DNA copy number by Longchamps RJ et al. **Figure S1.** The forward MR analyses: Plots of“leave-one-out” analyses for MR analyses of the causal effect of mtDNA copy number with the risk of cardiometabolic disease. (A) Obesity, (B) hypertension, (C) dyslipidemia, (D) T2DM, (E) CAD, (F) Stroke, (G) Ischemic stroke, (H) Heart failure. The horizontal lines in the figure represents beta value and its 95% confidence interval [CI] of causal inference, which indicates the genetic effect of the SNP on cardiometabolic disease. **Figure S2.** The reverse MR analyses: Casual effect of cardiometabolic disease on mtDNA copy number. Plots of “leave-one-out” analyses for MR analyses. (A) Obesity, (B) hypertension, (C) dyslipidemia, (D) T2DM, (E) CAD, (F) Stroke, (G) Ischemic stroke, (H) Heart failure. The horizontal lines in the figure represents beta value and its 95% confidence interval [CI] of causal inference, which indicates the genetic effect of the SNP on cardiometabolic disease. **Figure S3.** The forward MR analyses (validation analysis using mtDNA copy number by Longchamps): Scatter plot of the association between mtDNA copy number  and cardiometabolic disease. (A) Obesity, (B) hypertension, (C) dyslipidemia, (D) T2DM, (E) CAD, (F) Stroke, (G) Ischemic stroke, (H) Heart failure. The four methods applied in the current manuscript were all depicted. Lines in black, red, green, and blue represent IVW, MR‐Egger, weighted median, and weight mode methods. **Figure S4.** The forward MR analyses (validation analysis using mtDNA copy number by Longchamps). Plots of “leave-one-out” analyses for MR analyses. (A) Obesity, (B) hypertension, (C) dyslipidemia, (D) T2DM, (E) CAD, (F) Stroke, (G) Ischemic stroke, (H) Heart failure. The horizontal lines in the figure represents beta value and its 95% confidence interval [CI] of causal inference, which indicates the genetic effect of the SNP on cardiometabolic disease.

## Data Availability

Not applicable.

## Abbreviations

ARIC: Atherosclerosis Risk in Communities
CAD: Coronary artery disease
CARDIoGRAMplusC4D: The Coronary Artery Disease Genome-wide Replication and Meta-analysis Plus the Coronary Artery Disease Genetics
CHARGE: The Cohorts for Heart and Aging Research in Genomic Epidemiology
CHS: Cardiovascular Health Study
DIAGRAM: Diabetes Genetics Replication and Meta-analysis
GWAS: Genome-wide association study
IS: Ischemic stroke
IVW: Inverse variance weighting
LD: Linkage disequilibrum
mtDNA: Mitochondrial DNA
MR: Mendelian randomization
MR-PRESSO: Mendelian Randomization Pleiotropy RESidual Sum and Outlier
MI: Myocardial infarction
OR: Odds ratio
SNPs: Single nucleotide polymorphisms
STROBE-MR: Strengthening the Reporting of Observational Studies in Epidemiology Using Mendelian Randomization
T2DM: Type 2 diabetes
UKB: The UK Biobank
