# Supplementary material for: The role of mitochondrial DNA copy number in cardiometabolic disease: a bidirectional two-sample mendelian randomization study
Source: Cardiovasc Diabetol. 2024 Jan 28;23:45. doi: 10.1186/s12933-023-02074-1 (PMC10823732; doi:10.1186/s12933-023-02074-1)
Supplement: Supplementary file 1 — Additional file 1: Table S1. Genetic variants used as instrumental variables for mitochondrial DNA copy number. Table S2. Genetic variants used as instrumental variables for the cardiometabolic disease in the reverse MR analyses. Table S3. Genetic variants used as instrumental variables for mitochondrial DNA copy number by Longchamps RJ et al. Table S4. Replication analyses for the MR analyses on the forward associations of mitochondrial DNA copy number with cardiometabolic diseases using GWAS summary data of mitochondrial DNA copy number by Longchamps RJ et al. Figure S1. The forward MR analyses: Plots of“leave-one-out” analyses for MR analyses of the causal effect of mtDNA copy number with the risk of cardiometabolic disease. (A) Obesity, (B) hypertension, (C) dyslipidemia, (D) T2DM, (E) CAD, (F) Stroke, (G) Ischemic stroke, (H) Heart failure. The horizontal lines in the figure represents beta value and its 95% confidence interval [CI] of causal inference, which indicates the genetic effect of the SNP on cardiometabolic disease. Figure S2. The reverse MR analyses: Casual effect of cardiometabolic disease on mtDNA copy number. Plots of “leave-one-out” analyses for MR analyses. (A) Obesity, (B) hypertension, (C) dyslipidemia, (D) T2DM, (E) CAD, (F) Stroke, (G) Ischemic stroke, (H) Heart failure. The horizontal lines in the figure represents beta value and its 95% confidence interval [CI] of causal inference, which indicates the genetic effect of the SNP on cardiometabolic disease. Figure S3. The forward MR analyses (validation analysis using mtDNA copy number by Longchamps): Scatter plot of the association between mtDNA copy number and cardiometabolic disease. (A) Obesity, (B) hypertension, (C) dyslipidemia, (D) T2DM, (E) CAD, (F) Stroke, (G) Ischemic stroke, (H) Heart failure. The four methods applied in the current manuscript were all depicted. Lines in black, red, green, and blue represent IVW, MR‐Egger, weighted median, and weight mode methods. Figure S4. The fo [file 12933_2023_2074_MOESM1_ESM.docx]

Additional file 1

The role of mitochondrial DNA copy number in cardiometabolic disease: a bidirectional Mendelian randomization study

**Table S1.** Genetic variants used as instrumental variables for mitochondrial DNA copy number.

**Table S2.** Genetic variants used as instrumental variables for the cardiometabolic disease in the reverse MR analyses.

**Table S3.** Genetic variants used as instrumental variables for mitochondrial DNA copy number by Longchamps RJ et al.

**Table S4.** Replication analyses for the MR analyses on the forward associations of mitochondrial DNA copy number with cardiometabolic diseases using GWAS summary data of mitochondrial DNA copy number by Longchamps RJ et al.

[**Figure S1**](https://europepmc.org/articles/PMC9349767/figure/jmv28008-fig-0003/) The forward MR analyses: Plots of “leave-one-out” analyses for MR analyses of the causal effect of mtDNA copy number with the risk of cardiometabolic disease. (A) Obesity, (B) hypertension, (C) dyslipidemia, (D) T2DM, (E) CAD, (F) Stroke, (G) Ischemic stroke, (H) Heart failure. The horizontal lines in the figure represents beta value and its 95% confidence interval [CI] of causal inference, which indicates the genetic effect of the SNP on cardiometabolic disease.

**Figure S2** The reverse MR analyses: Casual effect of cardiometabolic disease on mtDNA copy number. Plots of “leave-one-out” analyses for MR analyses. (A) Obesity, (B) hypertension, (C) dyslipidemia, (D) T2DM, (E) CAD, (F) Stroke, (G) Ischemic stroke, (H) Heart failure. The horizontal lines in the figure represents beta value and its 95% confidence interval [CI] of causal inference, which indicates the genetic effect of the SNP on cardiometabolic disease.

[**Figure S3**](https://europepmc.org/articles/PMC9349767/figure/jmv28008-fig-0003/) The forward MR analyses (validation analysis using mtDNA copy number by Longchamps): Scatter plot of the association between mtDNA copy number and cardiometabolic disease. (A) Obesity, (B) hypertension, (C) dyslipidemia, (D) T2DM, (E) CAD, (F) Stroke, (G) Ischemic stroke, (H) Heart failure. The four methods applied in the current manuscript were all depicted. Lines in black, red, green, and blue represent IVW, MR‐Egger, weighted median, and weight mode methods.

**Figure S4** The forward MR analyses (validation analysis using mtDNA copy number by Longchamps). Plots of “leave-one-out” analyses for MR analyses. (A) Obesity, (B) hypertension, (C) dyslipidemia, (D) T2DM, (E) CAD, (F) Stroke, (G) Ischemic stroke, (H) Heart failure. The horizontal lines in the figure represents beta value and its 95% confidence interval [CI] of causal inference, which indicates the genetic effect of the SNP on cardiometabolic disease.

**Table S1. Genetic variants used as instrumental variables for mitochondrial DNA copy number.**

| **SNP** | **Effect allele** | **Other allele** | **Beta** | **SE** | ***P* value** |
| --- | --- | --- | --- | --- | --- |
| rs114694170 | C | T | 0.0331 | 0.0045 | 3.04E-13 |
| rs2015599 | A | G | 0.0123 | 0.0021 | 6.03E-09 |
| rs2304693 | A | G | 0.0181 | 0.0028 | 5.11E-11 |
| rs74750282 | C | T | 0.038 | 0.0038 | 4.29E-24 |
| rs1065853 | T | G | 0.0388 | 0.0039 | 1.59E-23 |
| rs4698839 | T | C | 0.0124 | 0.0022 | 9.47E-09 |
| rs11064074 | T | C | 0.0197 | 0.0021 | 4.87E-20 |
| rs11553699 | G | A | 0.0445 | 0.0032 | 1.45E-43 |
| rs12148 | G | T | -0.0139 | 0.0022 | 1.32E-10 |
| rs6959832 | A | G | -0.021 | 0.0021 | 2.10E-23 |
| rs2263663 | T | C | 0.02 | 0.0024 | 1.97E-16 |
| rs12247015 | G | A | 0.0337 | 0.0021 | 1.28E-55 |
| rs1613662 | A | G | -0.0167 | 0.0028 | 3.90E-09 |
| rs1569419 | C | T | 0.0189 | 0.0025 | 6.87E-14 |
| rs289713 | A | T | -0.0149 | 0.0027 | 4.14E-08 |
| rs10835540 | A | T | 0.0184 | 0.0023 | 5.74E-16 |
| rs11085147 | T | C | 0.0756 | 0.0036 | 1.54E-95 |
| rs10749636 | A | G | 0.0155 | 0.0025 | 5.43E-10 |
| rs192890685 | T | C | 0.0247 | 0.0044 | 1.93E-08 |
| rs77261872 | T | C | 0.0262 | 0.0032 | 2.28E-16 |
| rs56069439 | A | C | -0.0274 | 0.0023 | 2.30E-32 |
| rs701834 | T | C | -0.0254 | 0.0026 | 1.12E-22 |
| rs12426673 | T | G | -0.0141 | 0.0021 | 4.03E-11 |
| rs2038480 | T | A | 0.0162 | 0.0027 | 1.39E-09 |
| rs200309755 | T | C | -0.018 | 0.0022 | 1.91E-16 |
| rs4284061 | A | T | -0.0186 | 0.0022 | 2.97E-17 |
| rs74874677 | G | A | -0.0819 | 0.0071 | 3.62E-31 |
| rs342293 | G | C | 0.0283 | 0.0021 | 1.05E-40 |
| rs17850455 | G | C | 0.091 | 0.0104 | 1.81E-18 |
| rs12052715 | G | C | -0.0133 | 0.0024 | 1.72E-08 |
| rs4814776 | A | C | -0.03 | 0.0023 | 2.00E-40 |
| rs72660908 | G | C | 0.0457 | 0.0021 | 8.25E-101 |
| rs5745582 | T | C | 0.021 | 0.0028 | 3.46E-14 |
| rs13088724 | A | G | 0.0175 | 0.0026 | 7.70E-12 |
| rs1354034 | C | T | -0.0268 | 0.0022 | 2.05E-35 |
| rs1127787 | A | G | -0.0159 | 0.0028 | 1.49E-08 |
| rs11764390 | A | G | -0.0122 | 0.0022 | 1.55E-08 |
| rs2977608 | C | A | 0.0236 | 0.0025 | 2.58E-21 |
| rs1760940 | C | A | 0.0263 | 0.0024 | 5.20E-27 |
| rs3766744 | A | G | -0.0179 | 0.0021 | 2.91E-17 |
| rs2274319 | C | T | -0.0139 | 0.0022 | 4.09E-10 |
| rs182346769 | A | G | 0.0213 | 0.0027 | 7.87E-15 |
| rs5012419 | G | A | 0.0247 | 0.0022 | 2.66E-29 |
| rs2290507 | A | G | 0.0223 | 0.0031 | 3.64E-13 |
| rs28665408 | C | A | 0.0157 | 0.0021 | 2.11E-13 |
| rs6105852 | A | G | 0.0221 | 0.0021 | 1.41E-25 |
| rs212930 | G | A | 0.0149 | 0.0025 | 3.18E-09 |
| rs2245946 | A | G | 0.0317 | 0.0023 | 9.38E-45 |
| rs13084580 | T | C | 0.0244 | 0.0033 | 2.19E-13 |
| rs2322718 | G | T | 0.0136 | 0.0021 | 1.53E-10 |
| rs62641680 | A | G | -0.0903 | 0.0063 | 4.63E-47 |
| rs1598010 | A | G | 0.0168 | 0.003 | 2.41E-08 |
| rs78909033 | A | G | -0.021 | 0.0031 | 1.00E-11 |
| rs72698722 | T | C | -0.0192 | 0.0027 | 1.25E-12 |
| rs156355 | C | T | 0.0214 | 0.0022 | 6.41E-23 |
| rs385893 | C | T | 0.0153 | 0.0021 | 5.96E-13 |
| rs57066921 | G | T | -0.1081 | 0.0081 | 8.98E-41 |
| rs9425601 | A | G | 0.0125 | 0.0023 | 3.04E-08 |
| rs3961455 | A | G | 0.0142 | 0.0024 | 4.31E-09 |
| rs4895441 | G | A | 0.0177 | 0.0024 | 8.31E-14 |
| rs445 | T | C | 0.0207 | 0.0036 | 8.85E-09 |
| rs7705526 | A | C | 0.0178 | 0.0023 | 6.46E-15 |

Abbreviations: SE, Standard Error; SNPs, single nucleotide polymorphisms.

**Table S2.** **Genetic variants used as instrumental variables for the cardiometabolic disease in the reverse MR analyses.**

| **SNP** | **Effect allele** | **Other allele** | **EAF** | **Beta** | **SE** | ***P* value** | **Diseases/Traits** |
| --- | --- | --- | --- | --- | --- | --- | --- |
| rs10145461 | G | T | 0.520141 | 0.0639401 | 0.010909 | 4.59E-09 | Obesity |
| rs10938398 | A | G | 0.472256 | 0.0789173 | 0.0108227 | 3.06E-13 | Obesity |
| rs10973268 | G | C | 0.549772 | -0.063146 | 0.0108715 | 6.31E-09 | Obesity |
| rs11030104 | G | A | 0.166009 | -0.102882 | 0.0148579 | 4.38E-12 | Obesity |
| rs11184961 | G | A | 0.629768 | 0.0628573 | 0.0112437 | 2.27E-08 | Obesity |
| rs12639495 | C | T | 0.173283 | 0.0799697 | 0.0141726 | 1.68E-08 | Obesity |
| rs12967878 | C | T | 0.181886 | 0.118835 | 0.0137104 | 4.42E-18 | Obesity |
| rs12986742 | C | T | 0.41663 | 0.0609516 | 0.0109632 | 2.70E-08 | Obesity |
| rs13394970 | G | T | 0.545199 | -0.0609925 | 0.0108863 | 2.11E-08 | Obesity |
| rs141145395 | C | T | 0.585172 | 0.0645386 | 0.0110198 | 4.72E-09 | Obesity |
| rs1429935 | C | A | 0.492494 | -0.0591985 | 0.0108426 | 4.77E-08 | Obesity |
| rs1445594 | G | A | 0.333537 | -0.0672044 | 0.0115887 | 6.67E-09 | Obesity |
| rs186989 | G | A | 0.762415 | 0.071888 | 0.0128614 | 2.28E-08 | Obesity |
| rs2867131 | C | T | 0.837203 | 0.130109 | 0.0151012 | 6.95E-18 | Obesity |
| rs34783010 | T | G | 0.258408 | -0.0883633 | 0.012567 | 2.05E-12 | Obesity |
| rs3798519 | C | A | 0.212738 | 0.0943248 | 0.0129894 | 3.82E-13 | Obesity |
| rs45551238 | T | C | 0.0489876 | -0.159641 | 0.0261753 | 1.07E-09 | Obesity |
| rs5758181 | A | G | 0.233948 | -0.0732154 | 0.0130018 | 1.79E-08 | Obesity |
| rs62395884 | T | C | 0.302975 | 0.0667543 | 0.0117435 | 1.31E-08 | Obesity |
| rs67553175 | A | G | 0.208168 | 0.106002 | 0.0131329 | 6.94E-16 | Obesity |
| rs6759321 | T | G | 0.347451 | -0.0700369 | 0.0114839 | 1.07E-09 | Obesity |
| rs7187776 | G | A | 0.43212 | 0.0639637 | 0.0108841 | 4.18E-09 | Obesity |
| rs7230605 | C | T | 0.172886 | 0.0823654 | 0.0141014 | 5.19E-09 | Obesity |
| rs8051717 | G | A | 0.296773 | 0.0649774 | 0.0117845 | 3.51E-08 | Obesity |
| rs9928094 | G | A | 0.428454 | 0.191146 | 0.0108026 | 4.63E-70 | Obesity |
| rs1021956 | T | G | 0.857585 | -0.0541302 | 0.00930344 | 5.95E-09 | Hypertension |
| rs11206828 | G | A | 0.641039 | -0.0397655 | 0.00678085 | 4.51E-09 | Hypertension |
| rs12035330 | G | A | 0.246895 | 0.0432368 | 0.00755244 | 1.04E-08 | Hypertension |
| rs12509595 | C | T | 0.313808 | 0.0881447 | 0.00702603 | 4.21E-36 | Hypertension |
| rs12749040 | A | G | 0.0813737 | 0.070513 | 0.0118389 | 2.58E-09 | Hypertension |
| rs1274960 | C | T | 0.454808 | -0.0365029 | 0.00653664 | 2.35E-08 | Hypertension |
| rs1275977 | A | G | 0.594416 | -0.085473 | 0.00661949 | 3.84E-38 | Hypertension |
| rs13032431 | T | G | 0.0740333 | 0.0732642 | 0.012502 | 4.62E-09 | Hypertension |
| rs13112725 | C | G | 0.828087 | 0.0499205 | 0.00868906 | 9.18E-09 | Hypertension |
| rs1317181 | T | G | 0.209068 | 0.0652439 | 0.00798069 | 2.95E-16 | Hypertension |
| rs1371617 | G | A | 0.661592 | 0.0402019 | 0.00689175 | 5.43E-09 | Hypertension |
| rs143439093 | G | A | 0.0754896 | -0.108612 | 0.0125789 | 5.90E-18 | Hypertension |
| rs146781867 | A | G | 0.135291 | 0.0562804 | 0.00954497 | 3.72E-09 | Hypertension |
| rs16846073 | G | C | 0.143516 | -0.0562877 | 0.00928534 | 1.34E-09 | Hypertension |
| rs1879053 | C | T | 0.548733 | -0.0419901 | 0.00654278 | 1.38E-10 | Hypertension |
| rs1912813 | A | G | 0.0857131 | -0.0659537 | 0.011693 | 1.70E-08 | Hypertension |
| rs2643826 | T | C | 0.403982 | 0.0452613 | 0.00663766 | 9.18E-12 | Hypertension |
| rs268263 | A | T | 0.793553 | 0.0443267 | 0.00806132 | 3.83E-08 | Hypertension |
| rs2704368 | G | A | 0.836926 | -0.0639276 | 0.00881169 | 4.02E-13 | Hypertension |
| rs34319137 | T | C | 0.527126 | -0.0425865 | 0.00650754 | 5.98E-11 | Hypertension |
| rs34502618 | G | A | 0.15525 | 0.0525011 | 0.00895766 | 4.60E-09 | Hypertension |
| rs3771612 | A | G | 0.866248 | -0.0538492 | 0.00952484 | 1.57E-08 | Hypertension |
| rs3774573 | C | T | 0.240217 | -0.0428465 | 0.00762985 | 1.96E-08 | Hypertension |
| rs3790604 | A | C | 0.170586 | 0.109004 | 0.00861683 | 1.12E-36 | Hypertension |
| rs3821843 | A | G | 0.660314 | 0.0403534 | 0.00694517 | 6.24E-09 | Hypertension |
| rs60595656 | C | T | 0.685573 | -0.0415617 | 0.00704014 | 3.56E-09 | Hypertension |
| rs62248283 | A | G | 0.214791 | -0.0434112 | 0.00795035 | 4.75E-08 | Hypertension |
| rs62271373 | A | T | 0.04343 | 0.0955535 | 0.0159301 | 1.99E-09 | Hypertension |
| rs6818149 | C | A | 0.642736 | -0.0382093 | 0.00676823 | 1.65E-08 | Hypertension |
| rs72689147 | T | G | 0.217554 | -0.0635194 | 0.00788414 | 7.84E-16 | Hypertension |
| rs7644186 | A | C | 0.251086 | -0.0420313 | 0.00754302 | 2.52E-08 | Hypertension |
| rs7683733 | T | C | 0.338674 | 0.0406763 | 0.00688846 | 3.53E-09 | Hypertension |
| rs880315 | C | T | 0.412582 | 0.0734027 | 0.00660693 | 1.12E-28 | Hypertension |
| rs9851392 | A | T | 0.550572 | 0.0381184 | 0.00653091 | 5.33E-09 | Hypertension |
| rs10455872 | G | A | 0.0462472 | 0.184682 | 0.0231664 | 1.56E-15 | Dyslipidemia |
| rs114165349 | C | G | 0.0374386 | 0.151373 | 0.0258812 | 4.95E-09 | Dyslipidemia |
| rs115478735 | T | A | 0.198234 | 0.0979976 | 0.0124672 | 3.83E-15 | Dyslipidemia |
| rs11591147 | T | G | 0.0362203 | -0.444579 | 0.0301744 | 3.92E-49 | Dyslipidemia |
| rs117733303 | G | A | 0.0108998 | 0.293955 | 0.0455478 | 1.09E-10 | Dyslipidemia |
| rs12740374 | T | G | 0.214849 | -0.14492 | 0.0125537 | 7.92E-31 | Dyslipidemia |
| rs12916 | C | T | 0.453249 | 0.0823152 | 0.0101226 | 4.23E-16 | Dyslipidemia |
| rs1367117 | A | G | 0.280077 | 0.104015 | 0.0110792 | 6.09E-21 | Dyslipidemia |
| rs149603090 | T | G | 0.0408334 | 0.188918 | 0.0246211 | 1.68E-14 | Dyslipidemia |
| rs1556516 | C | G | 0.421157 | 0.0866961 | 0.0101733 | 1.57E-17 | Dyslipidemia |
| rs185567543 | T | A | 0.0217319 | 0.311016 | 0.0324844 | 1.03E-21 | Dyslipidemia |
| rs1883711 | C | G | 0.0621576 | 0.148733 | 0.0203621 | 2.78E-13 | Dyslipidemia |
| rs2738447 | C | A | 0.605963 | 0.0638717 | 0.0103679 | 7.25E-10 | Dyslipidemia |
| rs2954021 | G | A | 0.536006 | -0.092603 | 0.0100806 | 4.07E-20 | Dyslipidemia |
| rs3005923 | A | G | 0.0260254 | -0.25515 | 0.0342967 | 1.01E-13 | Dyslipidemia |
| rs34130316 | T | C | 0.196492 | -0.0774439 | 0.0128467 | 1.66E-09 | Dyslipidemia |
| rs4246215 | T | G | 0.443048 | -0.0572833 | 0.0101685 | 1.77E-08 | Dyslipidemia |
| rs4959027 | G | A | 0.84396 | 0.106842 | 0.0141621 | 4.55E-14 | Dyslipidemia |
| rs499883 | A | G | 0.528457 | 0.102179 | 0.0101807 | 1.05E-23 | Dyslipidemia |
| rs646356 | A | T | 0.831304 | 0.0974878 | 0.0137589 | 1.39E-12 | Dyslipidemia |
| rs73015011 | C | T | 0.10263 | -0.266382 | 0.017555 | 5.25E-52 | Dyslipidemia |
| rs7412 | T | C | 0.0536479 | -0.440391 | 0.0246089 | 1.28E-71 | Dyslipidemia |
| rs77645768 | A | G | 0.0268217 | 0.269671 | 0.0296925 | 1.06E-19 | Dyslipidemia |
| rs780094 | C | T | 0.64492 | -0.0627353 | 0.0105212 | 2.48E-09 | Dyslipidemia |
| rs964184 | C | G | 0.855523 | -0.159514 | 0.0139564 | 2.98E-30 | Dyslipidemia |
| rs9753033 | T | A | 0.0818478 | -0.158307 | 0.0190708 | 1.03E-16 | Dyslipidemia |
| rs3768321 | G | T | 0.2 | 0.075 | 0.0094 | 9.60E-16 | T2DM |
| rs58432198 | C | T | 0.12 | -0.073 | 0.012 | 1.40E-09 | T2DM |
| rs6657819 | G | C | 0.53 | -0.042 | 0.0075 | 1.70E-08 | T2DM |
| rs2793829 | C | T | 0.11 | 0.095 | 0.012 | 1.40E-15 | T2DM |
| rs340874 | T | C | 0.55 | 0.071 | 0.0075 | 4.80E-21 | T2DM |
| rs2494196 | C | A | 0.28 | -0.061 | 0.0083 | 2.20E-13 | T2DM |
| rs348330 | G | A | 0.64 | -0.052 | 0.0078 | 3.70E-11 | T2DM |
| rs1260326 | T | C | 0.6 | 0.067 | 0.0076 | 7.40E-19 | T2DM |
| rs77101426 | G | A | 0.071 | -0.16 | 0.015 | 5.50E-28 | T2DM |
| rs243019 | T | C | 0.46 | 0.059 | 0.0075 | 2.10E-15 | T2DM |
| rs2080385 | G | T | 0.24 | -0.052 | 0.0087 | 1.80E-09 | T2DM |
| rs11688682 | G | C | 0.27 | -0.065 | 0.0088 | 1.60E-13 | T2DM |
| rs7579654 | C | T | 0.064 | -0.092 | 0.015 | 1.80E-09 | T2DM |
| rs10184004 | C | T | 0.41 | -0.08 | 0.0076 | 4.30E-26 | T2DM |
| rs113414093 | G | A | 0.051 | 0.12 | 0.02 | 6.60E-09 | T2DM |
| rs2972144 | A | G | 0.64 | 0.1 | 0.0078 | 2.90E-40 | T2DM |
| rs17036160 | C | T | 0.12 | -0.12 | 0.011 | 8.60E-26 | T2DM |
| rs35352848 | T | C | 0.21 | -0.089 | 0.0092 | 5.40E-22 | T2DM |
| rs7640397 | C | T | 0.58 | 0.046 | 0.0075 | 5.60E-10 | T2DM |
| rs2292662 | C | T | 0.16 | -0.067 | 0.01 | 8.30E-11 | T2DM |
| rs4132228 | C | T | 0.29 | -0.068 | 0.0083 | 2.60E-16 | T2DM |
| rs11708067 | A | G | 0.23 | -0.11 | 0.0089 | 3.10E-33 | T2DM |
| rs4679370 | T | C | 0.54 | 0.047 | 0.0075 | 3.40E-10 | T2DM |
| rs2347252 | T | C | 0.16 | 0.062 | 0.01 | 1.10E-09 | T2DM |
| rs114863656 | T | C | 0.04 | -0.13 | 0.02 | 1.40E-11 | T2DM |
| rs6770420 | G | A | 0.37 | -0.075 | 0.0077 | 2.60E-22 | T2DM |
| rs6780171 | T | A | 0.31 | 0.12 | 0.008 | 2.40E-51 | T2DM |
| rs4686471 | T | C | 0.61 | 0.07 | 0.0077 | 1.10E-19 | T2DM |
| rs56187241 | C | T | 0.041 | 0.13 | 0.019 | 7.30E-12 | T2DM |
| rs4865436 | C | G | 0.29 | 0.058 | 0.0088 | 3.50E-11 | T2DM |
| rs1046317 | T | C | 0.67 | 0.095 | 0.008 | 1.90E-32 | T2DM |
| rs10471048 | G | C | 0.65 | -0.045 | 0.0078 | 8.00E-09 | T2DM |
| rs7660000 | C | T | 0.29 | -0.046 | 0.0083 | 3.70E-08 | T2DM |
| rs6813195 | C | T | 0.28 | -0.063 | 0.0084 | 7.00E-14 | T2DM |
| rs73875816 | T | C | 0.15 | -0.071 | 0.011 | 2.00E-11 | T2DM |
| rs6885132 | C | G | 0.1 | -0.095 | 0.013 | 6.60E-14 | T2DM |
| rs2648731 | G | A | 0.22 | 0.051 | 0.009 | 1.50E-08 | T2DM |
| rs4865796 | G | A | 0.69 | 0.061 | 0.0081 | 6.70E-14 | T2DM |
| rs465002 | C | T | 0.74 | 0.08 | 0.0085 | 7.60E-21 | T2DM |
| rs4976033 | A | G | 0.41 | 0.047 | 0.0077 | 1.00E-09 | T2DM |
| rs4457053 | G | A | 0.69 | -0.069 | 0.0081 | 1.50E-17 | T2DM |
| rs115505614 | C | T | 0.05 | 0.19 | 0.018 | 3.80E-27 | T2DM |
| rs329124 | A | G | 0.42 | 0.055 | 0.0075 | 1.70E-13 | T2DM |
| rs3934712 | T | C | 0.21 | 0.051 | 0.0092 | 3.20E-08 | T2DM |
| rs648795 | A | T | 0.58 | -0.048 | 0.0076 | 3.50E-10 | T2DM |
| rs9379084 | G | A | 0.11 | -0.12 | 0.012 | 3.80E-23 | T2DM |
| rs7451008 | T | C | 0.27 | 0.15 | 0.0083 | 7.00E-76 | T2DM |
| rs3131012 | T | C | 0.54 | 0.051 | 0.0075 | 7.90E-12 | T2DM |
| rs547454879 | A | G | 0.25 | 0.074 | 0.011 | 3.90E-11 | T2DM |
| rs9273531 | C | T | 0.11 | 0.11 | 0.013 | 4.20E-17 | T2DM |
| rs998584 | C | A | 0.48 | 0.055 | 0.0075 | 2.20E-13 | T2DM |
| rs4714704 | G | A | 0.27 | 0.05 | 0.0084 | 2.60E-09 | T2DM |
| rs719727 | A | G | 0.24 | -0.074 | 0.0087 | 2.40E-17 | T2DM |
| rs1573090 | T | G | 0.47 | -0.055 | 0.0076 | 6.10E-13 | T2DM |
| rs2982521 | A | T | 0.62 | -0.044 | 0.0077 | 8.80E-09 | T2DM |
| rs474513 | A | G | 0.48 | -0.051 | 0.0075 | 1.30E-11 | T2DM |
| rs4709746 | C | T | 0.13 | -0.074 | 0.011 | 3.50E-11 | T2DM |
| rs17168486 | C | T | 0.17 | 0.073 | 0.0098 | 7.50E-14 | T2DM |
| rs2215383 | T | C | 0.54 | 0.071 | 0.0075 | 1.70E-21 | T2DM |
| rs849135 | G | A | 0.49 | -0.1 | 0.0075 | 7.20E-41 | T2DM |
| rs878521 | G | A | 0.25 | 0.062 | 0.0087 | 8.70E-13 | T2DM |
| rs61462211 | G | C | 0.33 | 0.068 | 0.0082 | 1.00E-16 | T2DM |
| rs6459733 | G | C | 0.33 | -0.062 | 0.008 | 1.00E-14 | T2DM |
| rs17091891 | T | C | 0.12 | -0.076 | 0.012 | 6.80E-11 | T2DM |
| rs516946 | T | C | 0.76 | 0.097 | 0.0088 | 5.60E-28 | T2DM |
| rs1320164 | G | A | 0.5 | -0.051 | 0.0074 | 5.50E-12 | T2DM |
| rs13266634 | C | T | 0.31 | -0.12 | 0.0081 | 3.10E-53 | T2DM |
| rs17772814 | G | A | 0.082 | -0.082 | 0.015 | 4.00E-08 | T2DM |
| rs3757971 | T | C | 0.38 | 0.063 | 0.0078 | 8.20E-16 | T2DM |
| rs7015203 | T | C | 0.45 | -0.044 | 0.0075 | 5.20E-09 | T2DM |
| rs10974438 | A | C | 0.36 | 0.059 | 0.0078 | 3.20E-14 | T2DM |
| rs62563593 | A | G | 0.39 | 0.048 | 0.0076 | 3.20E-10 | T2DM |
| rs2383205 | A | G | 0.6 | 0.056 | 0.0076 | 1.60E-13 | T2DM |
| rs10811660 | G | A | 0.17 | -0.18 | 0.01 | 5.90E-69 | T2DM |
| rs17791513 | A | G | 0.07 | -0.1 | 0.015 | 8.10E-12 | T2DM |
| rs2796441 | G | A | 0.41 | -0.08 | 0.0076 | 1.30E-25 | T2DM |
| rs28429551 | T | A | 0.75 | 0.079 | 0.0088 | 2.00E-19 | T2DM |
| rs11257655 | C | T | 0.21 | 0.095 | 0.009 | 7.70E-26 | T2DM |
| rs3847343 | G | A | 0.61 | 0.045 | 0.0078 | 1.10E-08 | T2DM |
| rs2642596 | C | T | 0.45 | 0.046 | 0.0075 | 1.00E-09 | T2DM |
| rs703967 | A | C | 0.46 | -0.08 | 0.0075 | 3.00E-26 | T2DM |
| rs34744311 | C | T | 0.37 | -0.12 | 0.0077 | 1.00E-57 | T2DM |
| rs116425039 | G | A | 0.01 | -0.31 | 0.04 | 1.10E-14 | T2DM |
| rs35011184 | G | A | 0.23 | 0.33 | 0.0089 | 1.00E-200 | T2DM |
| rs35198330 | T | G | 0.05 | 0.15 | 0.017 | 9.00E-18 | T2DM |
| rs72631105 | G | A | 0.19 | 0.058 | 0.0098 | 3.70E-09 | T2DM |
| rs35777422 | G | A | 0.35 | -0.043 | 0.0078 | 4.10E-08 | T2DM |
| rs76223293 | G | C | 0.19 | 0.055 | 0.0096 | 1.30E-08 | T2DM |
| rs4929965 | A | G | 0.62 | -0.07 | 0.0078 | 1.60E-19 | T2DM |
| rs231360 | C | T | 0.39 | 0.07 | 0.0077 | 1.80E-19 | T2DM |
| rs2237895 | A | C | 0.42 | 0.11 | 0.0076 | 9.90E-47 | T2DM |
| rs5215 | C | T | 0.63 | -0.079 | 0.0077 | 9.40E-25 | T2DM |
| rs4923543 | G | A | 0.33 | 0.043 | 0.0079 | 4.50E-08 | T2DM |
| rs591291 | C | T | 0.3 | 0.058 | 0.0081 | 7.30E-13 | T2DM |
| rs3918296 | C | G | 0.03 | -0.14 | 0.024 | 1.80E-08 | T2DM |
| rs77464186 | A | C | 0.16 | -0.13 | 0.01 | 9.00E-37 | T2DM |
| rs10830963 | C | G | 0.28 | 0.1 | 0.0083 | 2.80E-36 | T2DM |
| rs3019209 | C | T | 0.68 | 0.046 | 0.008 | 8.30E-09 | T2DM |
| rs10893829 | T | C | 0.15 | -0.061 | 0.011 | 7.00E-09 | T2DM |
| rs4937325 | T | C | 0.72 | -0.047 | 0.0083 | 1.80E-08 | T2DM |
| rs11819995 | C | T | 0.22 | 0.055 | 0.0089 | 8.50E-10 | T2DM |
| rs4238013 | C | T | 0.79 | -0.07 | 0.0093 | 4.10E-14 | T2DM |
| rs76895963 | T | G | 0.02 | -0.52 | 0.031 | 1.30E-64 | T2DM |
| rs11048447 | G | T | 0.33 | 0.044 | 0.0079 | 2.70E-08 | T2DM |
| rs10842994 | C | T | 0.2 | -0.077 | 0.0094 | 2.00E-16 | T2DM |
| rs2258238 | A | T | 0.11 | 0.12 | 0.012 | 1.90E-21 | T2DM |
| rs1796330 | G | C | 0.43 | -0.051 | 0.0075 | 1.00E-11 | T2DM |
| rs1426371 | G | A | 0.26 | -0.051 | 0.0086 | 3.50E-09 | T2DM |
| rs1169299 | T | C | 0.47 | 0.061 | 0.0075 | 3.00E-16 | T2DM |
| rs7132277 | C | T | 0.19 | -0.053 | 0.0096 | 2.50E-08 | T2DM |
| rs10773051 | C | T | 0.23 | -0.053 | 0.0089 | 2.60E-09 | T2DM |
| rs34584161 | A | G | 0.24 | -0.051 | 0.0088 | 5.20E-09 | T2DM |
| rs963740 | A | T | 0.29 | -0.046 | 0.0082 | 1.40E-08 | T2DM |
| rs1359790 | G | A | 0.28 | -0.091 | 0.0083 | 6.50E-28 | T2DM |
| rs17122772 | C | G | 0.22 | 0.052 | 0.0091 | 8.00E-09 | T2DM |
| rs8017808 | G | T | 0.25 | -0.048 | 0.0087 | 3.70E-08 | T2DM |
| rs12912777 | C | T | 0.12 | 0.085 | 0.012 | 3.30E-13 | T2DM |
| rs11638890 | G | T | 0.35 | 0.047 | 0.0079 | 3.40E-09 | T2DM |
| rs8037894 | G | C | 0.43 | -0.047 | 0.0075 | 4.40E-10 | T2DM |
| rs28661116 | A | G | 0.56 | -0.045 | 0.0075 | 1.50E-09 | T2DM |
| rs4886876 | C | T | 0.75 | 0.076 | 0.0086 | 9.80E-19 | T2DM |
| rs28678152 | T | C | 0.74 | -0.079 | 0.0085 | 1.90E-20 | T2DM |
| rs8037137 | T | C | 0.13 | 0.072 | 0.011 | 2.90E-11 | T2DM |
| rs6600191 | T | C | 0.18 | -0.065 | 0.0098 | 3.80E-11 | T2DM |
| rs9930501 | A | G | 0.44 | 0.054 | 0.0075 | 6.00E-13 | T2DM |
| rs72802342 | C | A | 0.078 | -0.16 | 0.014 | 2.70E-29 | T2DM |
| rs8063007 | C | A | 0.15 | -0.058 | 0.011 | 2.70E-08 | T2DM |
| rs1076902 | C | T | 0.12 | 0.074 | 0.012 | 1.00E-09 | T2DM |
| rs2278524 | G | A | 0.3 | 0.056 | 0.0081 | 3.60E-12 | T2DM |
| rs1108646 | A | G | 0.67 | -0.05 | 0.008 | 4.40E-10 | T2DM |
| rs3094515 | T | C | 0.64 | 0.047 | 0.0085 | 4.20E-08 | T2DM |
| rs10908278 | T | A | 0.52 | -0.087 | 0.0075 | 2.60E-31 | T2DM |
| rs12454712 | T | C | 0.38 | -0.07 | 0.0079 | 5.60E-19 | T2DM |
| rs4804833 | A | G | 0.61 | -0.054 | 0.0077 | 1.60E-12 | T2DM |
| rs116843064 | G | A | 0.021 | -0.16 | 0.027 | 1.10E-08 | T2DM |
| rs12611068 | T | C | 0.6 | -0.055 | 0.0076 | 5.10E-13 | T2DM |
| rs10401969 | T | C | 0.08 | 0.12 | 0.014 | 7.70E-18 | T2DM |
| rs73927890 | C | T | 0.26 | -0.053 | 0.0085 | 3.90E-10 | T2DM |
| rs2238689 | T | C | 0.41 | 0.072 | 0.0076 | 1.40E-21 | T2DM |
| rs2747567 | A | G | 0.57 | 0.047 | 0.0078 | 1.30E-09 | T2DM |
| rs1800961 | C | T | 0.033 | 0.18 | 0.021 | 9.20E-18 | T2DM |
| rs1999536 | G | C | 0.57 | -0.047 | 0.0075 | 4.40E-10 | T2DM |
| rs13040225 | A | T | 0.47 | -0.056 | 0.0075 | 9.10E-14 | T2DM |
| rs736266 | A | T | 0.52 | 0.051 | 0.0075 | 1.00E-11 | T2DM |
| rs59944054 | G | A | 0.24 | 0.048 | 0.0088 | 4.60E-08 | T2DM |
| rs73883375 | A | G | 0.09 | -0.085 | 0.013 | 1.40E-10 | T2DM |
| rs116989257 | A | G | 0.06 | -0.1 | 0.017 | 2.90E-09 | T2DM |
| rs738408 | C | T | 0.22 | 0.062 | 0.0089 | 3.00E-12 | T2DM |
| rs28681372 | G | A | 0.6 | -0.047 | 0.0078 | 2.30E-09 | T2DM |
| rs9970807 | C | T | 0.016695 | 0.915097 | 0.12575 | 5.00E-14 | CAD |
| rs7528419 | A | G | 0.011482 | 0.78582 | 0.11453 | 1.97E-23 | CAD |
| rs6689306 | G | A | 0.0094061 | 0.552455 | -0.056012 | 2.60E-09 | CAD |
| rs67180937 | G | T | 0.0110551 | 0.663052 | 0.078807 | 1.01E-12 | CAD |
| rs16986953 | G | A | 0.0150265 | 0.895294 | -0.08516 | 1.45E-08 | CAD |
| rs515135 | C | T | 0.0121924 | 0.791985 | 0.067499 | 3.09E-08 | CAD |
| rs7568458 | T | A | 0.0095093 | 0.551482 | -0.059618 | 3.62E-10 | CAD |
| rs17678683 | T | G | 0.0166548 | 0.912319 | -0.098786 | 3.00E-09 | CAD |
| rs115654617 | C | A | 0.0158314 | 0.893038 | -0.137846 | 3.12E-18 | CAD |
| rs1199338 | A | C | 0.0124987 | 0.838134 | -0.073596 | 3.90E-09 | CAD |
| rs17087335 | G | T | 0.0111159 | 0.785363 | -0.060764 | 4.59E-08 | CAD |
| rs4593108 | C | G | 0.0115558 | 0.795349 | 0.07083 | 8.82E-10 | CAD |
| rs9349379 | A | G | 0.0096527 | 0.568394 | -0.131836 | 1.81E-42 | CAD |
| rs56336142 | T | C | 0.0118763 | 0.807262 | 0.066813 | 1.85E-08 | CAD |
| rs12202017 | A | G | 0.0099612 | 0.699953 | 0.066813 | 1.98E-11 | CAD |
| rs10080815 | T | G | 0.0308579 | 0.972442 | -0.246627 | 1.33E-15 | CAD |
| rs55730499 | C | T | 0.0242403 | 0.943757 | -0.316641 | 5.39E-39 | CAD |
| rs2107595 | G | A | 0.0112951 | 0.79953 | -0.073415 | 8.05E-11 | CAD |
| rs11556924 | C | T | 0.0110605 | 0.686675 | 0.072569 | 5.34E-11 | CAD |
| rs3918226 | C | T | 0.0221275 | 0.935485 | -0.133315 | 1.69E-09 | CAD |
| rs2891168 | A | G | 0.0091877 | 0.511332 | -0.193401 | 2.29E-98 | CAD |
| rs2519093 | C | T | 0.0117524 | 0.809128 | -0.079704 | 1.19E-11 | CAD |
| rs2487928 | G | A | 0.0095049 | 0.581779 | -0.062633 | 4.41E-11 | CAD |
| rs1870634 | G | T | 0.0097113 | 0.637485 | 0.075878 | 5.55E-15 | CAD |
| rs1412444 | C | T | 0.0096809 | 0.630869 | -0.066812 | 5.15E-12 | CAD |
| rs11191416 | T | G | 0.0135252 | 0.87253 | 0.079249 | 4.65E-09 | CAD |
| rs10840293 | A | G | 0.009619 | 0.549821 | 0.054714 | 1.28E-08 | CAD |
| rs2128739 | C | A | 0.0100568 | 0.676464 | -0.065565 | 7.05E-11 | CAD |
| rs2681472 | A | G | 0.0113331 | 0.798694 | -0.074114 | 6.17E-11 | CAD |
| rs11065979 | C | T | 0.0107672 | 0.634501 | -0.068556 | 1.93E-10 | CAD |
| rs11838776 | G | A | 0.0107552 | 0.736723 | -0.068566 | 1.83E-10 | CAD |
| rs10139550 | C | G | 0.0097569 | 0.576967 | -0.05538 | 1.38E-08 | CAD |
| rs56062135 | C | T | 0.0118937 | 0.794271 | 0.069743 | 4.52E-09 | CAD |
| rs4468572 | C | T | 0.0095277 | 0.585831 | 0.077234 | 4.44E-16 | CAD |
| rs8042271 | A | G | 0.0175662 | 0.097718 | -0.096711 | 3.68E-08 | CAD |
| rs7212798 | T | C | 0.0142216 | 0.853484 | -0.079961 | 1.88E-08 | CAD |
| rs663129 | G | A | 0.0105173 | 0.743165 | -0.058163 | 3.20E-08 | CAD |
| rs56289821 | G | A | 0.0170415 | 0.899622 | 0.13361 | 4.44E-15 | CAD |
| rs4420638 | A | G | 0.0140977 | 0.833964 | -0.091906 | 7.07E-11 | CAD |
| rs28451064 | G | A | 0.015952 | 0.878814 | -0.127571 | 1.33E-15 | CAD |
| rs180803 | T | G | 0.0283062 | 0.029268 | -0.180923 | 1.64E-10 | CAD |
| rs17035646 | G | A | 0.3514 | 0.0522 | 0.0096 | 2.70E-08 | stroke |
| rs11587860 | G | C | 0.3545 | -0.0689 | 0.0098 | 1.03E-12 | stroke |
| rs2129977 | A | G | 0.7881 | -0.0833 | 0.0111 | 3.08E-14 | stroke |
| rs11242678 | C | T | 0.2551 | 0.0643 | 0.0105 | 4.57E-10 | stroke |
| rs35276016 | C | T | 0.0851 | 0.0984 | 0.0184 | 4.45E-08 | stroke |
| rs2107595 | G | A | 0.1671 | 0.0803 | 0.0121 | 1.61E-11 | stroke |
| rs1537375 | T | C | 0.5021 | 0.0519 | 0.0091 | 5.88E-09 | stroke |
| rs10883926 | G | A | 0.6028 | 0.0503 | 0.0094 | 4.37E-08 | stroke |
| rs2284665 | G | T | 0.2145 | -0.0602 | 0.0111 | 2.92E-08 | stroke |
| rs470928 | G | A | 0.8683 | -0.0758 | 0.0137 | 1.58E-08 | stroke |
| rs10774624 | G | A | 0.5285 | -0.0654 | 0.0094 | 1.73E-12 | stroke |
| rs4942561 | G | T | 0.7581 | 0.064 | 0.0107 | 1.11E-09 | stroke |
| rs17035646 | G | A | 0.405 | 0.0536 | 0.0088 | 5.61E-10 | Ischemic stroke |
| rs2842873 | C | T | 0.3955 | -0.0572 | 0.0087 | 2.44E-11 | Ischemic stroke |
| rs6847935 | A | T | 0.3257 | 0.0784 | 0.0096 | 1.59E-16 | Ischemic stroke |
| rs6825454 | T | C | 0.3078 | 0.0564 | 0.0092 | 4.38E-10 | Ischemic stroke |
| rs11957829 | A | G | 0.1761 | -0.0719 | 0.0124 | 3.35E-09 | Ischemic stroke |
| rs4959130 | G | A | 0.1372 | 0.0832 | 0.014 | 1.40E-09 | Ischemic stroke |
| rs2107595 | G | A | 0.2264 | 0.0759 | 0.0102 | 4.99E-14 | Ischemic stroke |
| rs42039 | C | T | 0.2277 | -0.0656 | 0.0113 | 3.21E-09 | Ischemic stroke |
| rs7859727 | C | T | 0.5355 | 0.0514 | 0.0084 | 4.71E-10 | Ischemic stroke |
| rs2005108 | C | T | 0.1281 | 0.08 | 0.0145 | 1.72E-08 | Ischemic stroke |
| rs7304841 | A | C | 0.407 | -0.0484 | 0.0089 | 2.69E-08 | Ischemic stroke |
| rs3184504 | T | C | 0.5479 | -0.0751 | 0.0098 | 9.06E-15 | Ischemic stroke |
| rs35436 | C | T | 0.3814 | -0.0495 | 0.0089 | 1.34E-08 | Ischemic stroke |
| rs9526212 | A | G | 0.7614 | 0.0615 | 0.0101 | 5.68E-10 | Ischemic stroke |
| rs339800 | C | T | 0.2934 | 0.0597 | 0.0111 | 3.76E-08 | Ischemic stroke |
| rs4932370 | G | A | 0.333 | 0.0519 | 0.0094 | 1.68E-08 | Ischemic stroke |
| rs12445022 | G | A | 0.3057 | 0.0609 | 0.0095 | 7.25E-11 | Ischemic stroke |
| rs9909858 | T | C | 0.188 | 0.0893 | 0.0162 | 1.77E-08 | Ischemic stroke |
| rs1053007 | A | G | 0.6506 | 0.0479 | 0.0087 | 1.84E-08 | Ischemic stroke |
| rs1556516 | C | G | 0.0078 | 0.4845 | 0.0622 | 1.57E-15 | Heart failure |
| rs17617337 | T | C | 0.0095 | 0.2208 | -0.0561 | 3.65E-09 | Heart failure |
| rs17042102 | A | G | 0.0121 | 0.115 | 0.1103 | 5.71E-20 | Heart failure |
| rs4746140 | C | G | 0.0109 | 0.154 | -0.0666 | 1.10E-09 | Heart failure |
| rs600038 | T | C | 0.0096 | 0.7909 | -0.0569 | 3.68E-09 | Heart failure |
| rs4766578 | A | T | 0.0079 | 0.5287 | -0.0433 | 4.90E-08 | Heart failure |
| rs56094641 | A | G | 0.008 | 0.5842 | -0.0454 | 1.21E-08 | Heart failure |
| rs55730499 | T | C | 0.0157 | 0.0694 | 0.1058 | 1.83E-11 | Heart failure |
| rs11745324 | A | G | 0.0095 | 0.2277 | -0.0528 | 2.35E-08 | Heart failure |
| rs4135240 | T | C | 0.0084 | 0.6589 | 0.0486 | 6.84E-09 | Heart failure |
| rs1510226 | T | C | 0.0285 | 0.9807 | -0.162 | 1.27E-08 | Heart failure |
| rs660240 | T | C | 0.0097 | 0.2128 | -0.0611 | 3.25E-10 | Heart failure |

Abbreviations: CAD, coronary artery disease; EAF, effect allele frequency; SE, Standard Error; SNPs, single nucleotide polymorphisms; T2DM, type 2 diabetes mellitus.

**Table S3. Genetic variants used as instrumental variables for mitochondrial DNA copy number by Longchamps RJ et al.**

| **SNP** | **Effect allele** | **Other allele** | **Beta** | **SE** | ***P* value** |
| --- | --- | --- | --- | --- | --- |
| rs1569419 | T | C | -0.023 | 0.0025 | 1.90E-19 |
| rs3818157 | G | A | 0.0212 | 0.0021 | 3.00E-24 |
| rs204071 | C | T | -0.0225 | 0.0042 | 2.80E-08 |
| 1:156455314 | CTT | C | 0.0145 | 0.0023 | 9.90E-11 |
| rs2145380 | G | C | 0.0117 | 0.0022 | 1.20E-07 |
| rs6425521 | C | A | -0.0217 | 0.0026 | 2.30E-17 |
| rs9425311 | G | T | 0.0134 | 0.0021 | 3.90E-10 |
| 1:205246482 | TTTTG | T | 0.0163 | 0.0022 | 1.50E-13 |
| rs10749636 | G | A | -0.0153 | 0.0025 | 1.10E-09 |
| rs655029 | G | A | 0.0141 | 0.0023 | 1.60E-09 |
| rs711244 | C | T | 0.0138 | 0.0021 | 1.80E-10 |
| rs2302643 | G | A | -0.0121 | 0.0021 | 7.10E-09 |
| rs151084028 | T | G | 0.0854 | 0.0145 | 3.80E-09 |
| rs865551 | C | G | 0.0159 | 0.0022 | 3.50E-13 |
| rs62641680 | G | A | 0.1072 | 0.0063 | 1.50E-65 |
| rs74874677 | A | G | 0.0998 | 0.007 | 6.00E-46 |
| rs12052715 | C | G | 0.0168 | 0.0023 | 3.30E-13 |
| rs147820690 | C | T | -0.1239 | 0.0206 | 3.80E-09 |
| rs78909033 | G | A | 0.0246 | 0.0031 | 4.40E-15 |
| rs13084580 | C | T | -0.0269 | 0.0033 | 4.20E-16 |
| rs6786055 | G | T | 0.0147 | 0.0022 | 2.70E-12 |
| rs1354034 | T | C | 0.0311 | 0.0021 | 3.40E-49 |
| rs6778131 | T | A | 0.012 | 0.0022 | 2.30E-08 |
| rs1420476 | T | A | -0.0232 | 0.0051 | 5.70E-06 |
| rs34894010 | C | G | -0.0375 | 0.0052 | 1.60E-12 |
| rs755492124 | TAAAG | T | -0.0138 | 0.0021 | 2.40E-11 |
| rs6894574 | T | C | 0.0124 | 0.0022 | 1.30E-08 |
| rs2736100 | C | A | 0.0173 | 0.0021 | 1.60E-16 |
| rs34592828 | G | A | -0.0322 | 0.0051 | 5.80E-11 |
| rs114694170 | T | C | -0.0366 | 0.0045 | 9.90E-16 |
| rs56116444 | T | G | 0.025 | 0.004 | 3.80E-10 |
| rs193541 | C | T | -0.013 | 0.0021 | 6.10E-10 |
| rs926326 | A | G | -0.0171 | 0.0025 | 1.20E-11 |
| rs2844484 | A | G | 0.0174 | 0.0022 | 3.90E-16 |
| rs45552734 | C | T | -0.0209 | 0.0032 | 7.30E-11 |
| rs511515 | A | G | -0.0209 | 0.0023 | 5.20E-21 |
| rs5745582 | C | T | -0.0256 | 0.0027 | 1.20E-21 |
| rs4895441 | A | G | -0.02 | 0.0024 | 9.50E-18 |
| rs7744765 | T | C | 0.0153 | 0.0021 | 3.10E-13 |
| rs200957609 | G | A | -0.1168 | 0.0214 | 7.60E-08 |
| rs35585318 | T | C | 0.0134 | 0.0021 | 5.30E-10 |
| rs6943701 | A | T | -0.0181 | 0.0028 | 2.50E-11 |
| rs17260734 | T | A | -0.0112 | 0.0021 | 7.10E-08 |
| rs445 | C | T | -0.0207 | 0.0035 | 3.00E-09 |
| rs139141690 | G | A | 0.0989 | 0.0156 | 2.00E-10 |
| rs342293 | C | G | -0.0345 | 0.0021 | 7.00E-61 |
| rs74750282 | T | C | -0.0478 | 0.0037 | 7.30E-37 |
| rs602616 | C | G | 0.0193 | 0.0037 | 3.20E-08 |
| rs10085457 | G | A | -0.0092 | 0.0022 | 3.40E-05 |
| rs3110823 | A | C | -0.0329 | 0.0028 | 5.90E-33 |
| rs7800558 | T | C | 0.0122 | 0.0021 | 1.20E-08 |
| rs117728810 | G | A | 0.0239 | 0.0046 | 8.70E-08 |
| 8:6701534 | ACTC | A | -0.0122 | 0.0021 | 4.30E-09 |
| rs4284061 | T | A | 0.0201 | 0.0022 | 6.30E-20 |
| rs4841132 | A | G | 0.0276 | 0.0037 | 6.10E-14 |
| 8:103218144 | ATTGCTATTATAAATAAGCTT | A | -0.0284 | 0.0043 | 1.30E-11 |
| rs6986601 | A | G | 0.0119 | 0.0021 | 7.70E-09 |
| rs385893 | T | C | -0.0143 | 0.0021 | 1.20E-12 |
| 9:4838587 | TAC | T | -0.0207 | 0.0033 | 4.30E-10 |
| rs7033052 | G | C | 0.0091 | 0.0021 | 1.70E-05 |
| rs12247015 | A | G | -0.0422 | 0.0021 | 4.40E-89 |
| rs3876 | C | T | 0.0293 | 0.0039 | 1.30E-13 |
| rs56356712 | T | C | -0.024 | 0.0047 | 2.90E-07 |
| rs181771244 | C | T | -0.0745 | 0.0139 | 6.20E-08 |
| rs7896518 | A | G | 0.0515 | 0.0021 | 1.90E-127 |
| rs73349121 | G | C | 0.1339 | 0.0081 | 4.30E-61 |
| rs7902510 | C | T | 0.0351 | 0.0025 | 1.60E-43 |
| rs11594179 | C | T | -0.022 | 0.0025 | 1.50E-18 |
| rs7080536 | G | A | 0.0323 | 0.0052 | 6.30E-10 |
| rs4910886 | G | T | -0.0226 | 0.0022 | 6.20E-25 |
| rs2241942 | G | A | 0.0138 | 0.0025 | 1.70E-08 |
| rs11235573 | T | C | 0.0112 | 0.0021 | 3.80E-08 |
| rs74472890 | T | C | 0.0266 | 0.0048 | 1.20E-08 |
| rs1362214 | A | G | -0.0242 | 0.0021 | 8.50E-31 |
| rs1127787 | G | A | 0.0188 | 0.0028 | 3.50E-11 |
| rs2015599 | G | A | -0.0121 | 0.0021 | 1.50E-08 |
| rs6580981 | G | A | -0.0142 | 0.0021 | 2.40E-11 |
| rs1716505 | C | G | 0.0133 | 0.0023 | 4.70E-09 |
| rs12426673 | G | T | 0.0144 | 0.0021 | 2.20E-11 |
| rs749140768 | AGGCACCTCTTCACAGGAC | A | -0.0289 | 0.0039 | 1.10E-14 |
| rs11553699 | A | G | -0.0508 | 0.0032 | 1.20E-57 |
| rs7987027 | T | C | -0.0111 | 0.0021 | 1.20E-07 |
| rs1760940 | A | C | -0.0271 | 0.0024 | 2.00E-29 |
| rs2771358 | T | C | 0.0125 | 0.0024 | 8.80E-08 |
| rs17477725 | C | G | 0.0099 | 0.0021 | 3.00E-06 |
| rs4427713 | T | C | 0.012 | 0.0021 | 5.90E-09 |
| rs117948349 | G | A | 0.0345 | 0.0059 | 2.50E-09 |
| rs59488041 | T | A | 0.0238 | 0.0031 | 2.50E-14 |
| rs261290 | T | C | -0.0126 | 0.0022 | 1.80E-08 |
| rs141227171 | G | C | 0.0926 | 0.0233 | 1.10E-04 |
| rs3087374 | C | A | -0.0237 | 0.0038 | 2.60E-10 |
| rs151234 | G | C | 0.0201 | 0.0031 | 7.80E-11 |
| rs289713 | T | A | 0.0188 | 0.0027 | 2.50E-12 |
| rs55823018 | C | T | 0.0131 | 0.0023 | 5.90E-09 |
| rs7213347 | G | C | 0.0132 | 0.0023 | 2.00E-09 |
| rs12451698 | A | G | 0.0195 | 0.0025 | 9.90E-16 |
| rs12601687 | G | A | -0.0301 | 0.0034 | 2.20E-19 |
| rs1967556 | T | G | -0.0229 | 0.0021 | 1.30E-27 |
| rs17850455 | C | G | -0.1205 | 0.0102 | 5.20E-33 |
| rs11867543 | C | T | -0.0185 | 0.003 | 5.50E-10 |
| rs680478 | C | T | 0.0165 | 0.0024 | 7.70E-12 |
| rs77261872 | C | T | -0.0315 | 0.0032 | 4.50E-23 |
| rs17758695 | C | T | 0.0426 | 0.0062 | 2.00E-12 |
| rs28665408 | A | C | -0.0202 | 0.0021 | 4.70E-21 |
| rs12955015 | C | A | 0.0411 | 0.0072 | 1.40E-08 |
| rs10411696 | T | G | 0.0117 | 0.0021 | 2.30E-08 |
| rs11085147 | C | T | -0.0915 | 0.0036 | 3.00E-141 |
| rs3218221 | G | A | 0.0961 | 0.0166 | 3.70E-09 |
| rs142158911 | G | A | -0.0175 | 0.0033 | 4.60E-08 |
| rs57843631 | C | T | -0.0554 | 0.0078 | 2.40E-12 |
| rs139891465 | C | T | -0.0489 | 0.0053 | 1.30E-20 |
| rs10419397 | G | A | 0.0323 | 0.0023 | 7.10E-46 |
| rs35586766 | G | A | -0.0376 | 0.0036 | 4.50E-26 |
| 19:19756073 | AGCC | A | -0.0234 | 0.0041 | 1.10E-08 |
| rs7412 | C | T | -0.0401 | 0.0039 | 1.30E-24 |
| rs11667430 | A | G | -0.009 | 0.0021 | 2.70E-05 |
| rs1613662 | G | A | 0.0199 | 0.0028 | 5.40E-13 |
| rs11668201 | A | T | 0.015 | 0.0027 | 9.60E-09 |
| rs11696739 | G | A | 0.0189 | 0.0022 | 7.90E-19 |
| rs156355 | T | C | -0.0249 | 0.0021 | 4.30E-32 |
| rs4814776 | C | A | 0.0362 | 0.0022 | 5.40E-59 |
| rs185387034 | A | G | 0.0723 | 0.0115 | 2.80E-10 |
| rs754169 | T | A | -0.0274 | 0.0021 | 1.20E-38 |
| rs76599088 | C | T | -0.0796 | 0.0082 | 1.90E-22 |
| rs2426092 | A | C | -0.0124 | 0.0021 | 3.90E-09 |
| rs577050795 | C | CA | -0.012 | 0.0021 | 7.10E-09 |
| rs2245947 | G | T | -0.039 | 0.0022 | 2.50E-68 |
| rs75107793 | G | A | -0.0302 | 0.0041 | 2.30E-13 |
| rs12148 | T | G | 0.0166 | 0.0022 | 5.70E-15 |
| rs3002416 | T | C | NA | NA | 4.67E-17 |
| rs377206363 | CTATT | C | NA | NA | 6.46E-22 |
| rs56008802 | A | G | NA | NA | 5.77E-09 |
| rs392020 | C | T | NA | NA | 1.47E-36 |

**Table S4. Replication analyses for the MR analyses on the forward associations of mitochondrial DNA copy number with cardiometabolic diseases using GWAS summary data of mitochondrial DNA copy number by Longchamps RJ et al.**

| **Outcomes** | **No of SNPs** | **IVW** | | **Weighted median** | | **MR-Egger (intercept)** | | **MR-PRESSO** | | **MR-Egger intercept (P value)** | **Cochran’s Q test (I^2^ %)** | **Q pval** |
| --- | --- | --- | --- | --- | --- | --- | --- | --- | --- | --- | --- | --- |
|  |  | **OR (95% CI)** | **P value** | **OR (95% CI)** | **P value** | **OR (95% CI)** | **P value** | **OR (95% CI)** | **P value** |  |  |  |
| Obesity | 92 | 0.952(0.811-1.118) | 0.550 | 0.958(0.782-1.175) | 0.682 | 0.936(0.679-1.291) | 0.690 | 0.952(0.792-1.113) | 0.552 | 0.905 | 700.91  (87.02) | 1.46E-05 |
| Hypertension | 16 | 0.860(0.686-1.078) | 0.191 | 0.950(0.754-1.196) | 0.661 | 1.027(0.684-1.541) | 0.900 | 0.860(0.634-1.086) | 0.211 | 0.321 | 25.81  (41.88) | 0.040 |
| Dyslipidemia | 92 | 0.813(0.593-1.114) | 0.197 | 1.026(0.850-1.238) | 0.791 | 0.868(0.462-1.633) | 0.662 | 0.942(0.787-1.097) | 0.451 | 0.814 | 700.91 (87.02) | 6.99E-95 |
| T2DM | 91 | 0.958(0.836-1.097) | 0.533 | 1.008(0.886-1.148) | 0.903 | 1.153(0.885-1.503) | 0.295 | 0.910(0.792-1.028) | 0.120 | 0.114 | 265.73  (66.13) | 2.99E-19 |
| CAD | 88 | 0.842(0.713-0.994) | 0.042 | 0.882(0.745-1.044) | 0.146 | 0.878(0.621-1.243) | 0.466 | 0.878(0.753-1.003) | 0.044 | 0.785 | 216.11  (59.74) | 5.66E-13 |
| Stroke | 88 | 0.900(0.792-1.022) | 0.105 | 0.863 (0.731-1.019) | 0.083 | 0.753(0.582-0.975) | 0.034 | 0.874(0.761-0.986) | 0.021 | 0.126 | 158.67  (45.16) | 2.16E-04 |
| Ischemic stroke | 86 | 0.937(0.824-1.064) | 0.315 | 0.941(0.802-1.104) | 0.454 | 0.846(0.643-1.112) | 0.234 | 0.893(0.785-1.001) | 0.042 | 0.411 | 151.58  (43.92) | 1.22E-05 |
| Heart failure | 88 | 0.986 (0.892-1.091) | 0.791 | 0.967(0.846-1.105) | 0.625 | 0.925(0.754-1.137) | 0.463 | 0.981(0.894-1.068) | 0.663 | 0.487 | 120.32 (27.69) | 0.010 |

Abbreviations: CAD, coronary artery disease; CIs, 95% confidence intervals; MR-PRESSO, Mendelian randomization pleiotropy residual sum and outlier; SNPs, single nucleotide polymorphisms; T2DM, type 2 diabetes mellitus.

B

A


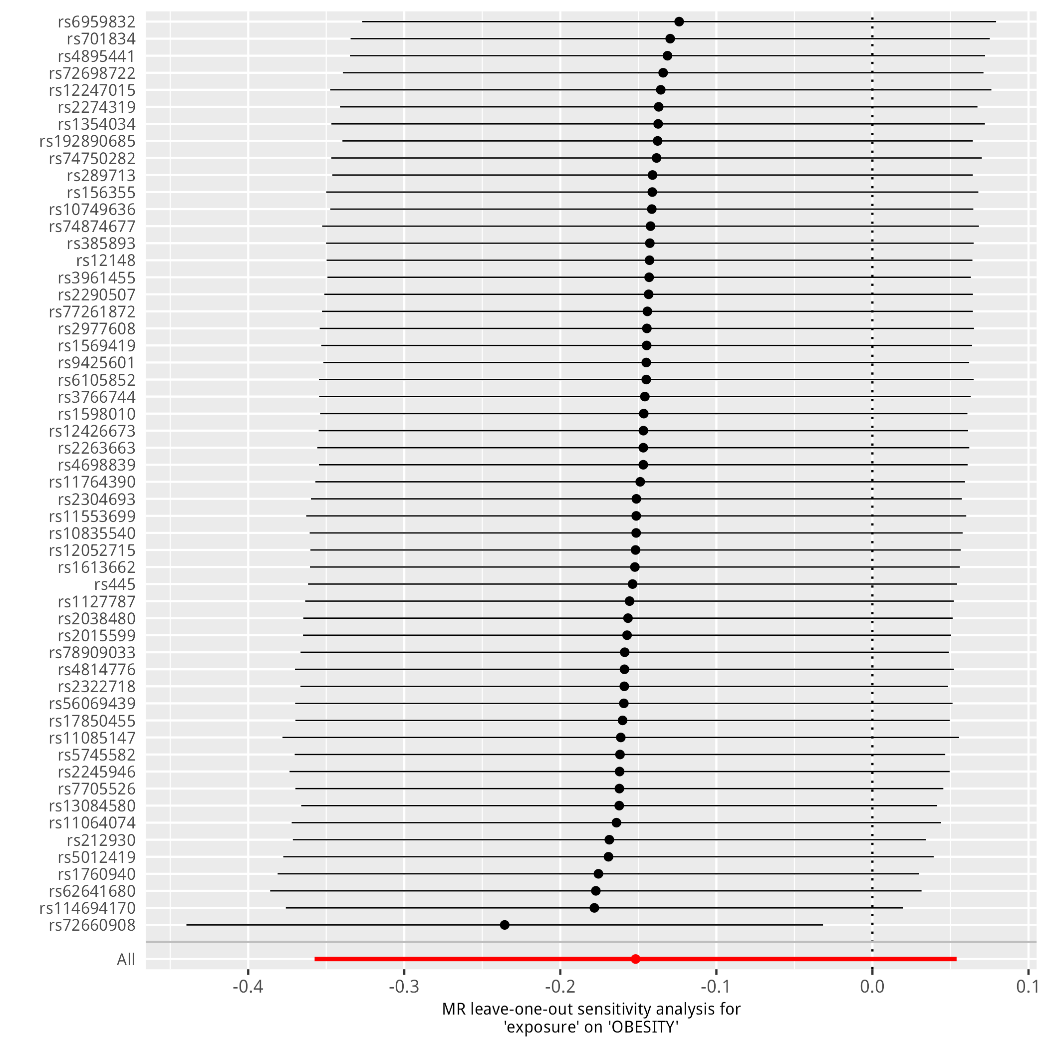

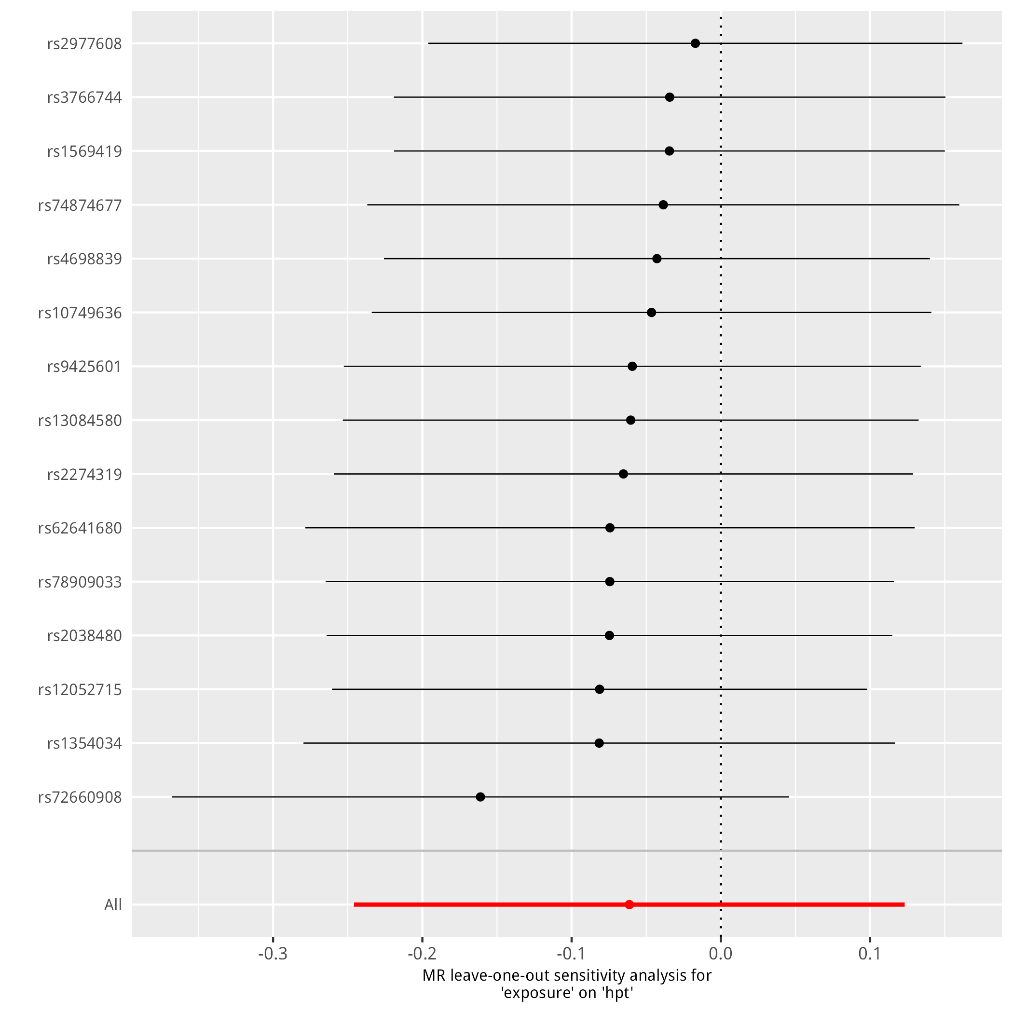


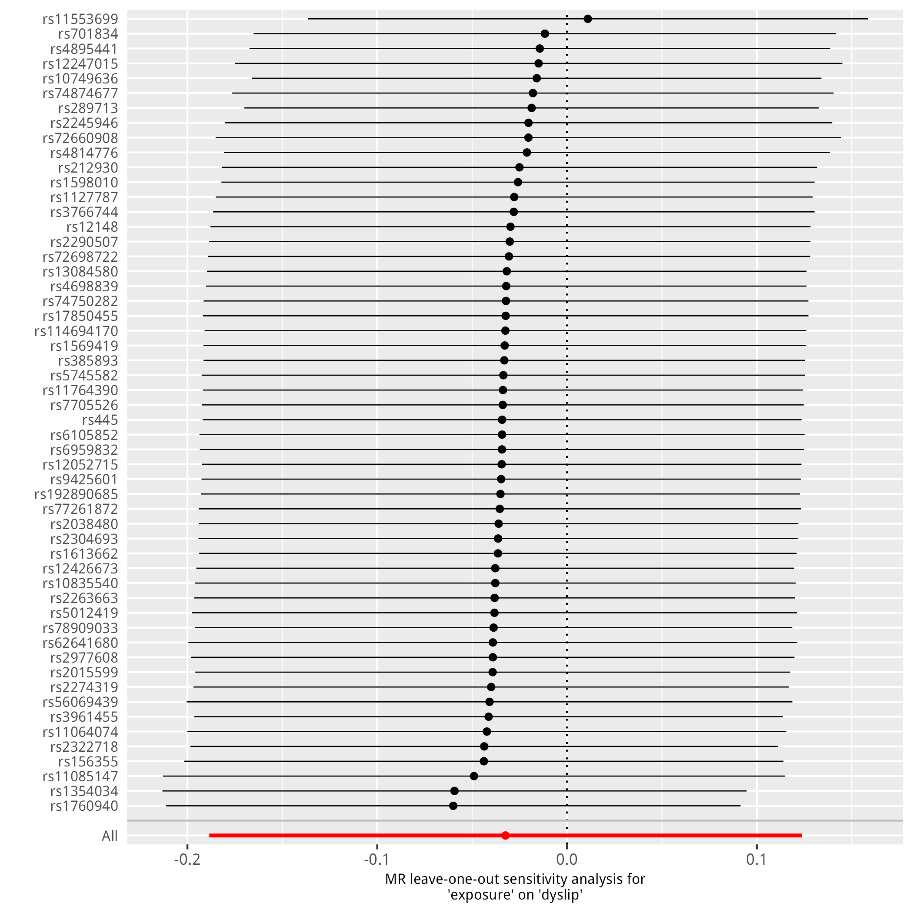

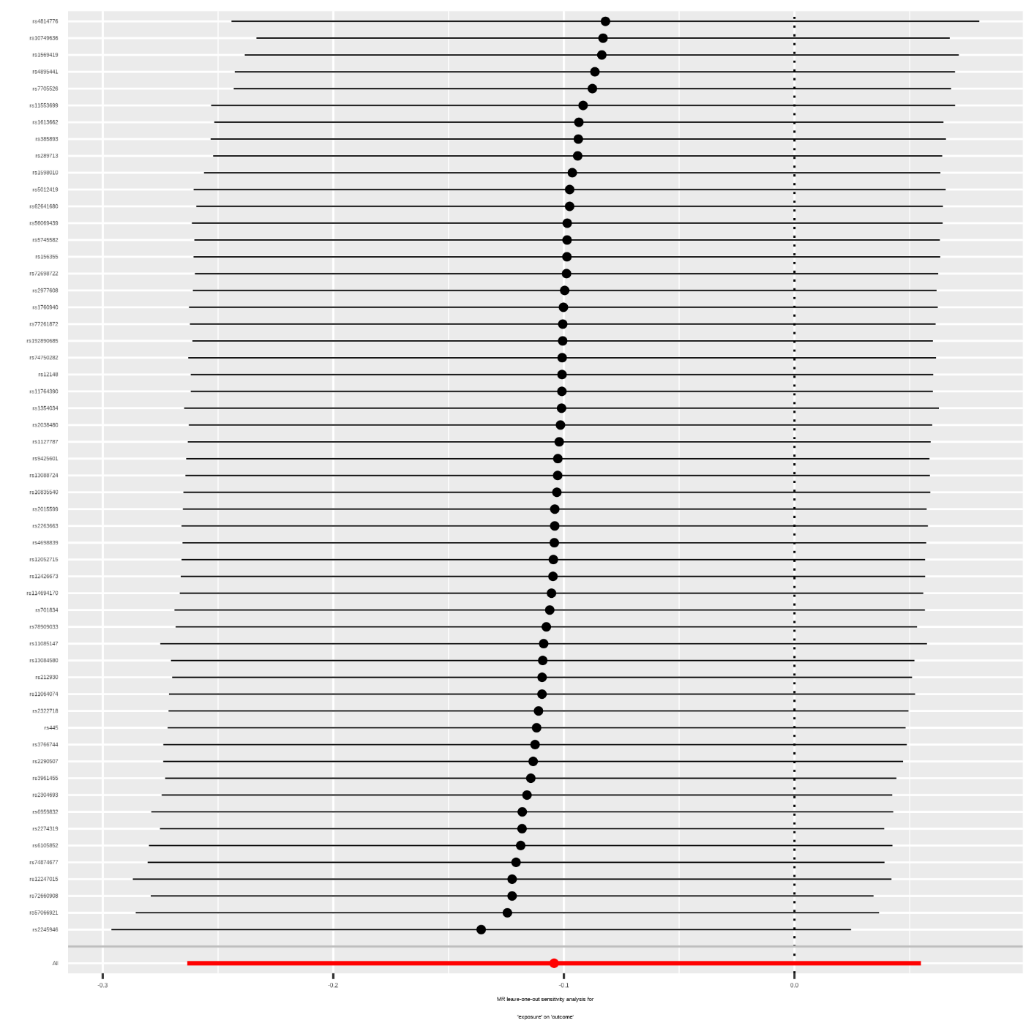


D

C


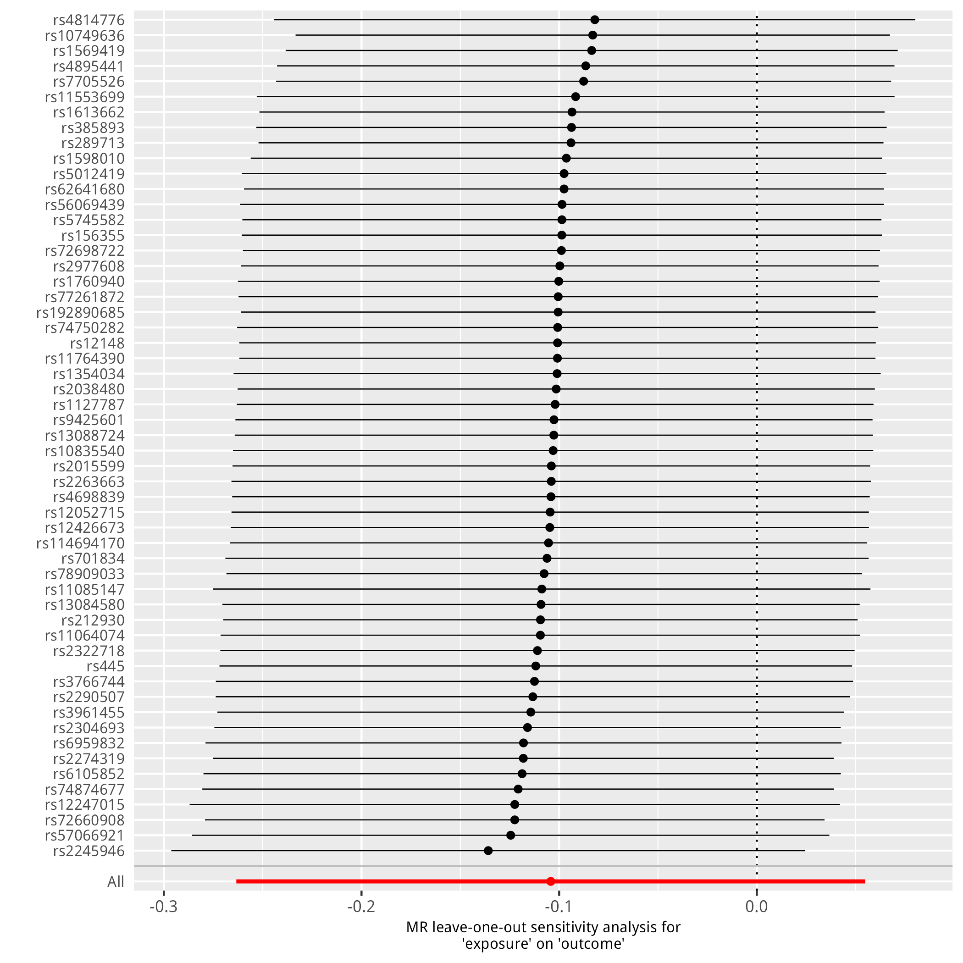

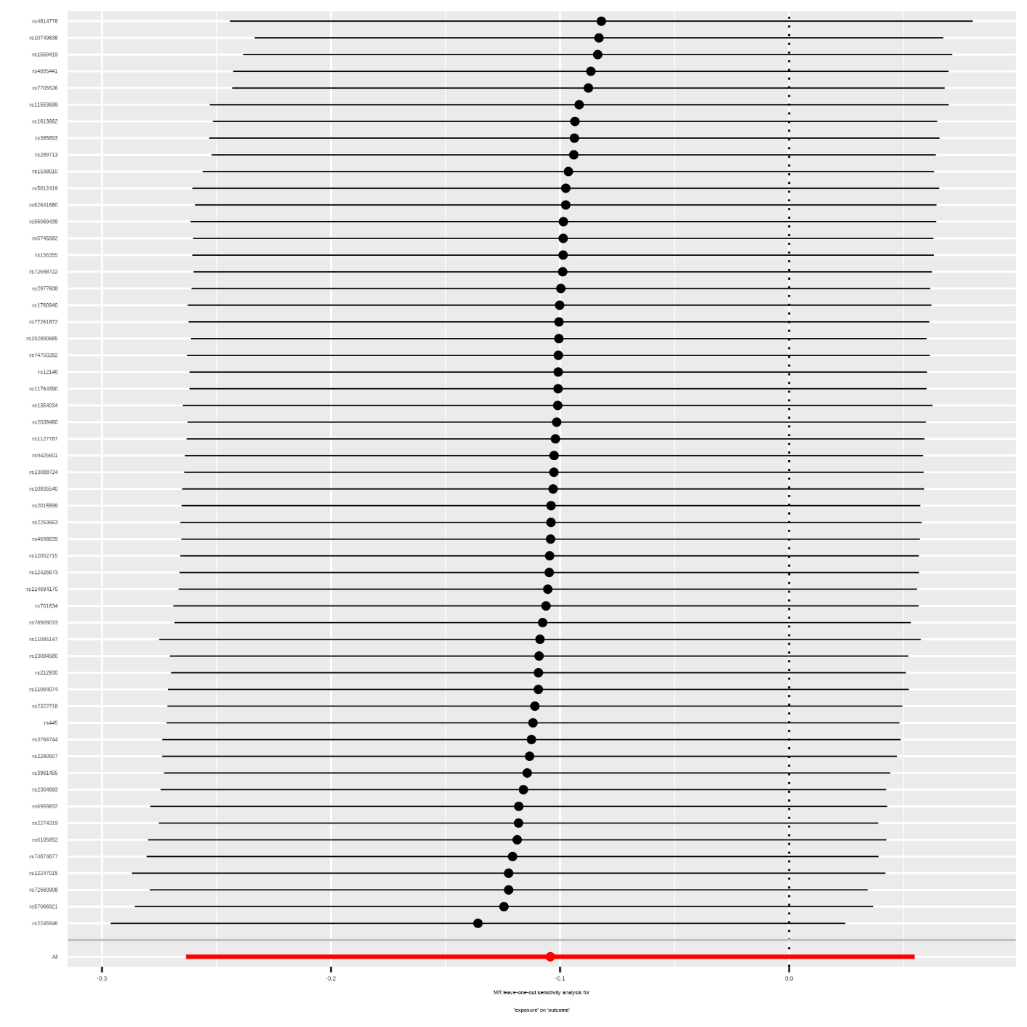


F

E


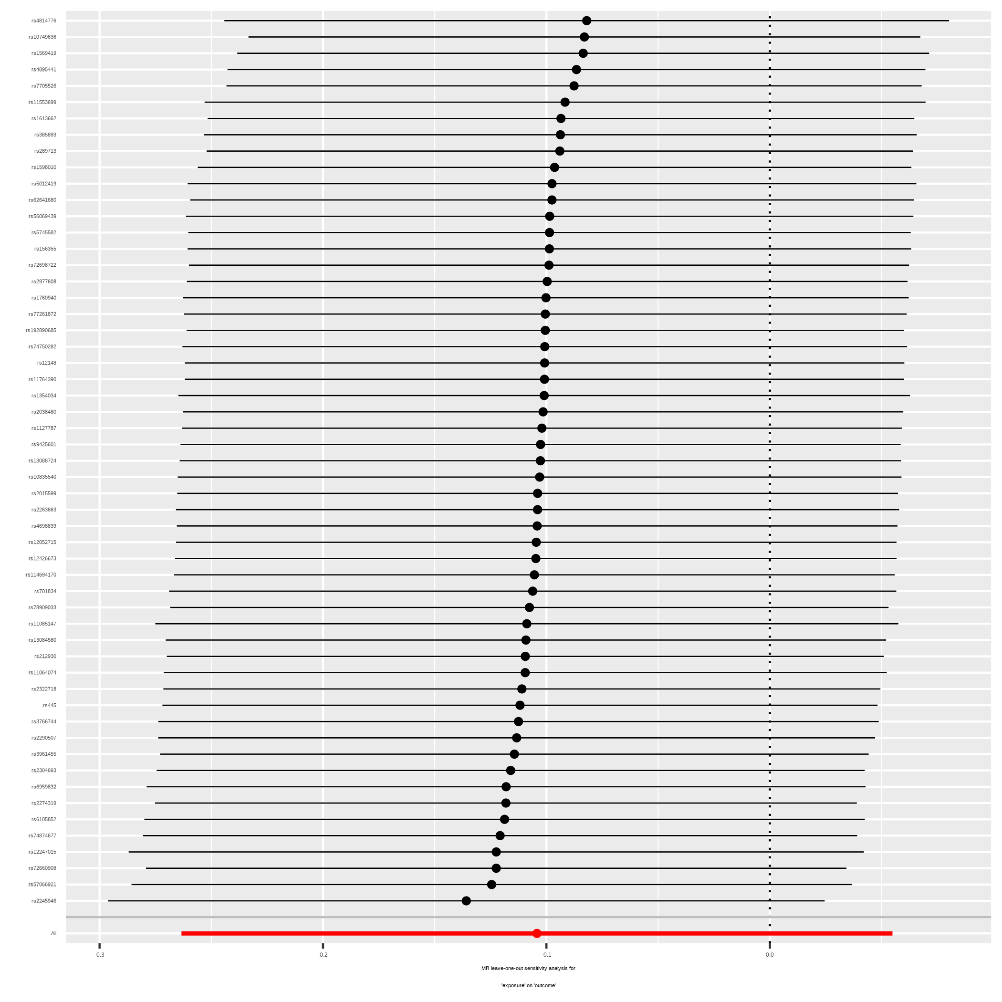

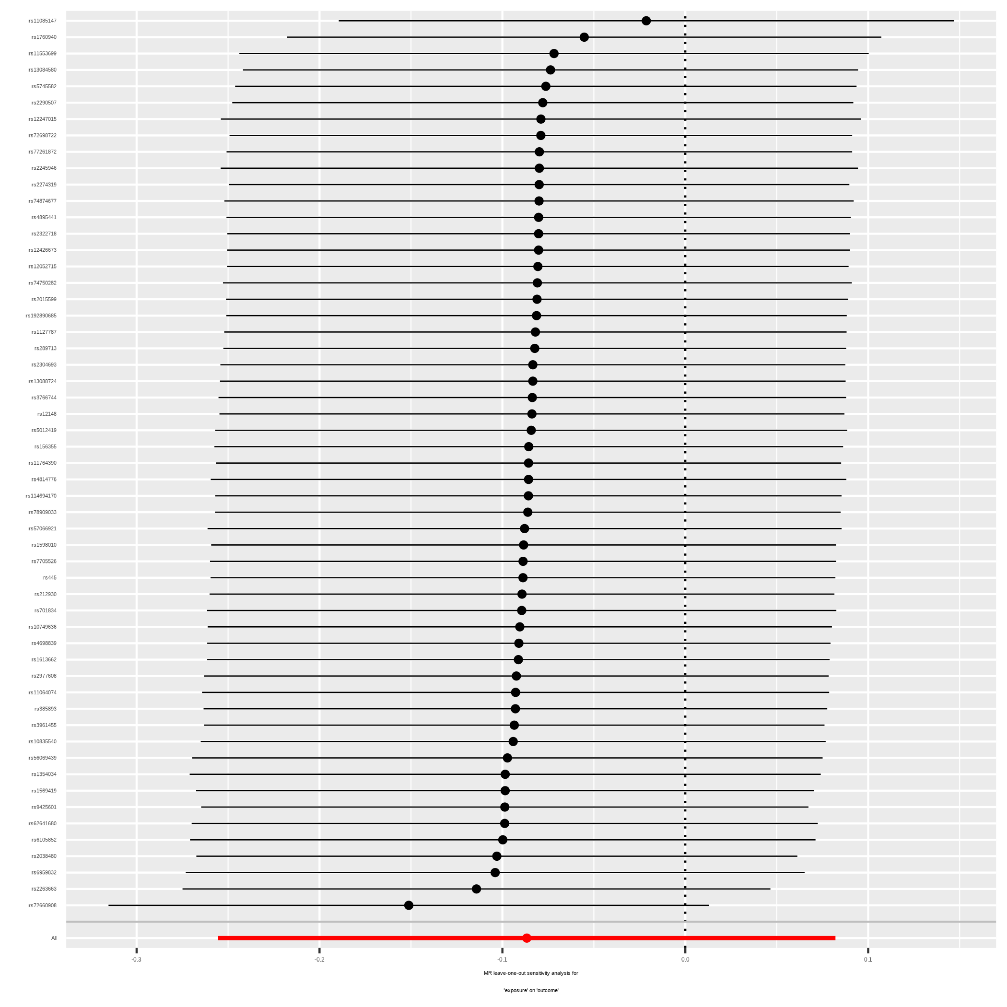


H

G

[**Figure S1**](https://europepmc.org/articles/PMC9349767/figure/jmv28008-fig-0003/) **The forward MR analyses:** **Plots of “leave-one-out” analyses for MR analyses of the causal effect of mtDNA copy number with the risk of cardiometabolic disease.** (A) Obesity, (B) hypertension, (C) dyslipidemia, (D) T2DM, (E) CAD, (F) Stroke, (G) Ischemic stroke, (H) Heart failure. The horizontal lines in the figure represents beta value and its 95% confidence interval [CI] of causal inference, which indicates the genetic effect of the SNP on cardiometabolic disease.

B

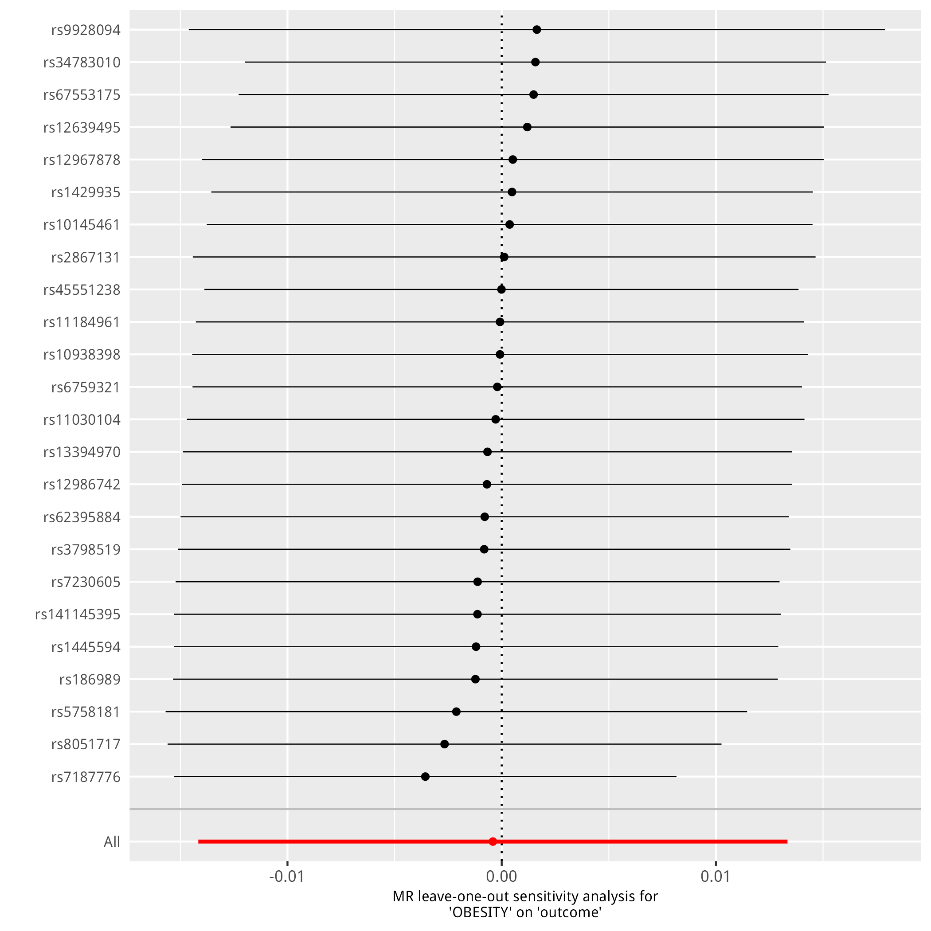

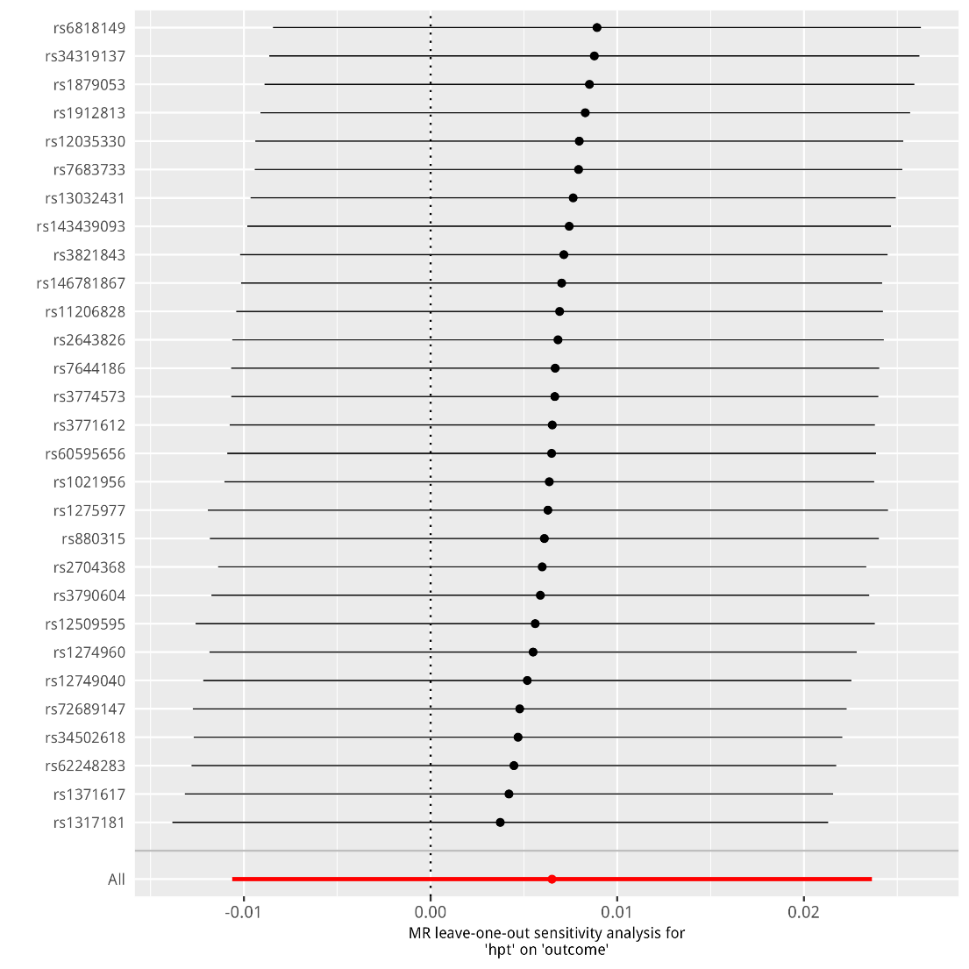


A

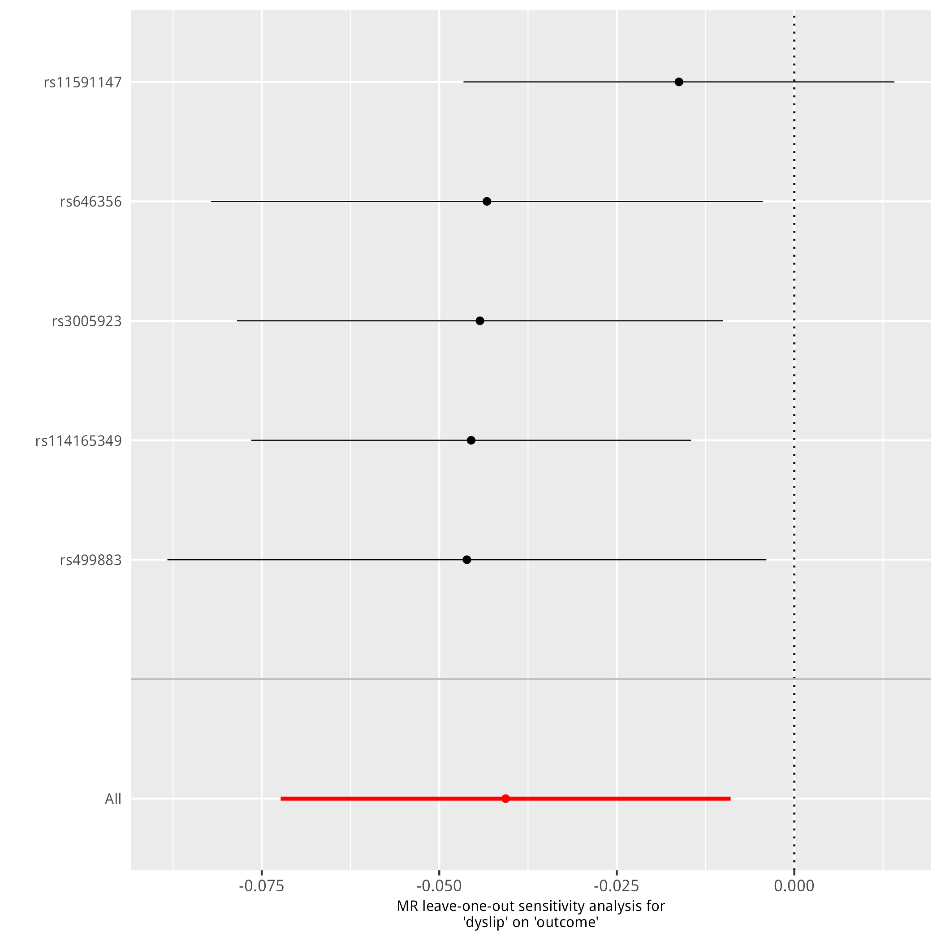

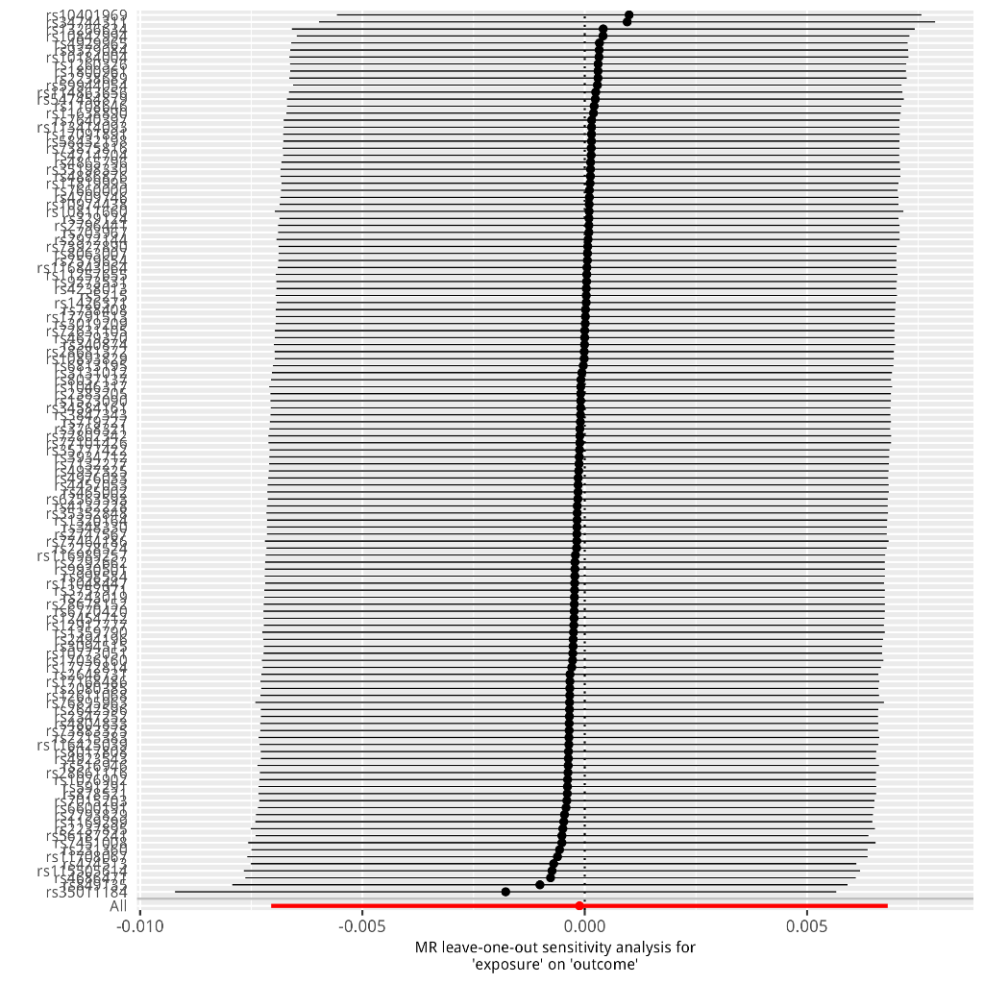


D

C

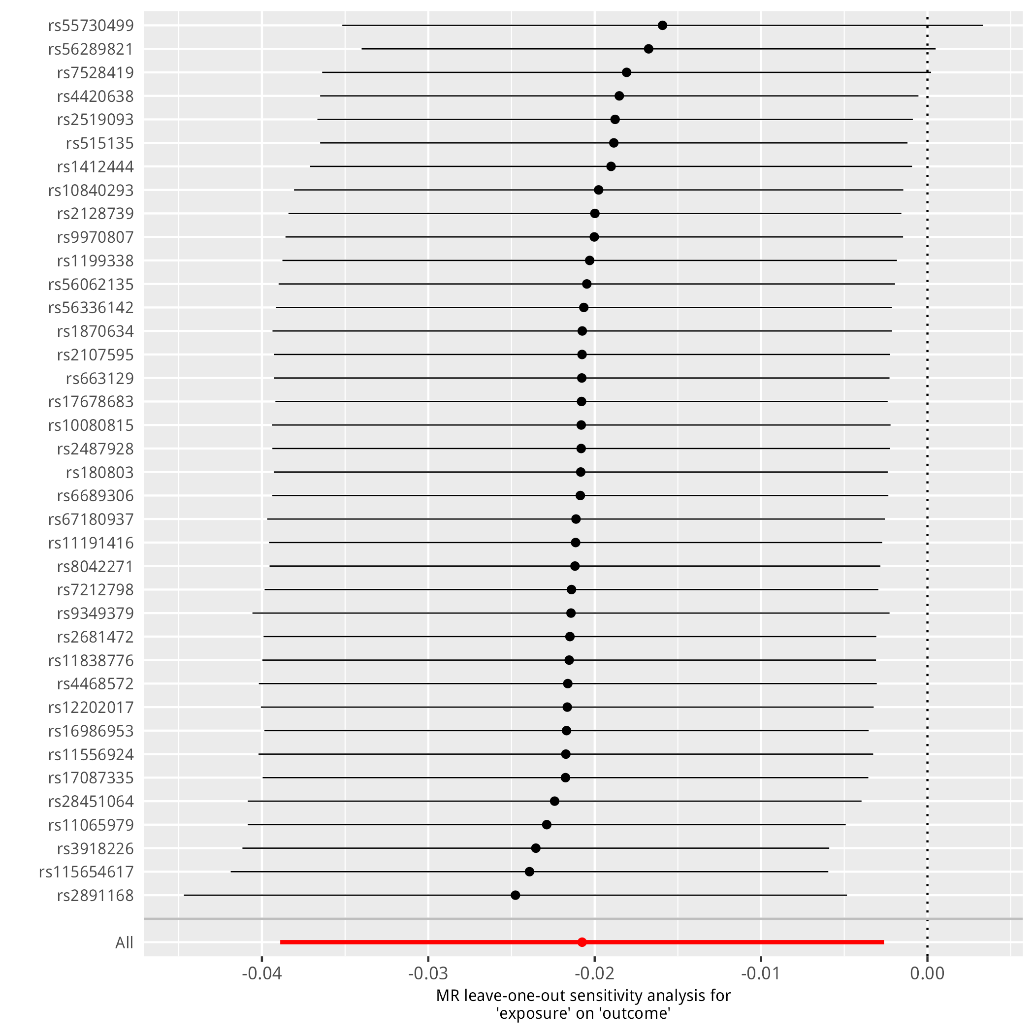

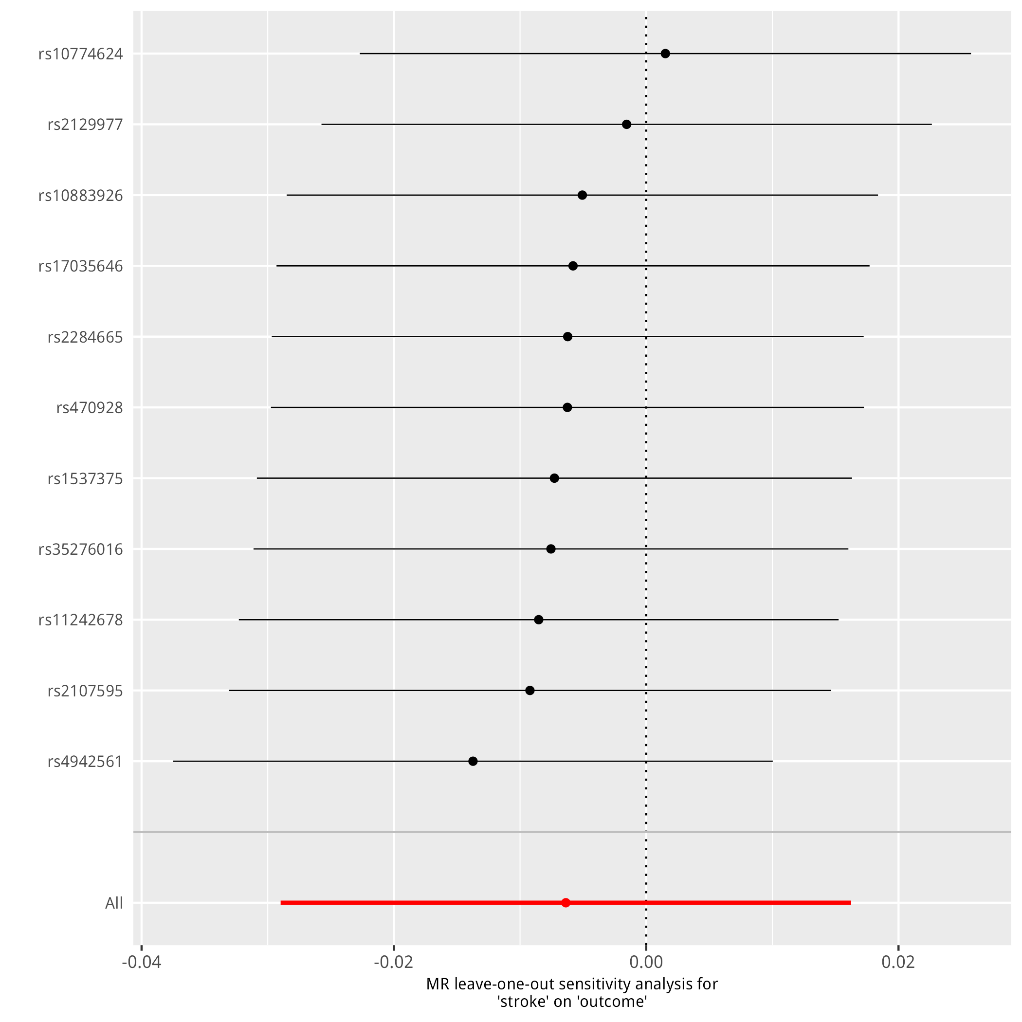


F

E

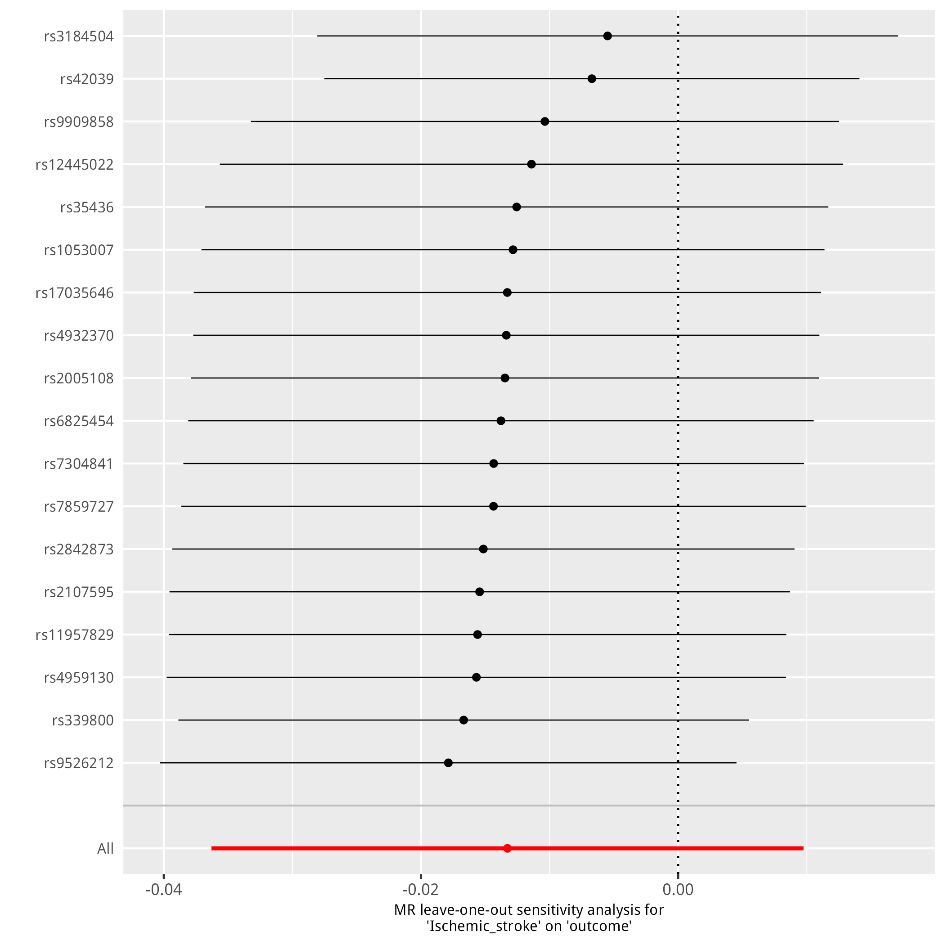

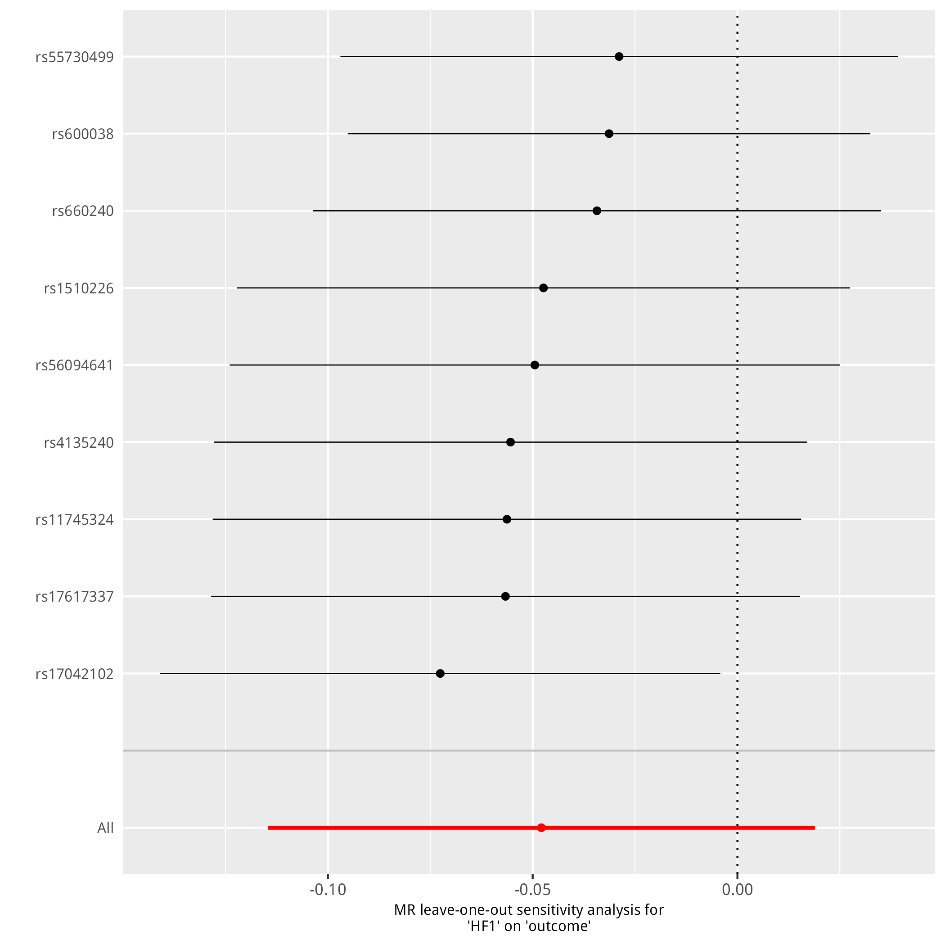


G

HE

**Figure S2 The reverse MR analyses: Casual effect of cardiometabolic disease on mtDNA copy number.** Plots of “leave-one-out” analyses for MR analyses. (A) Obesity, (B) hypertension, (C) dyslipidemia, (D) T2DM, (E) CAD, (F) Stroke, (G) Ischemic stroke, (H) Heart failure. The horizontal lines in the figure represents beta value and its 95% confidence interval [CI] of causal inference, which indicates the genetic effect of the SNP on cardiometabolic disease


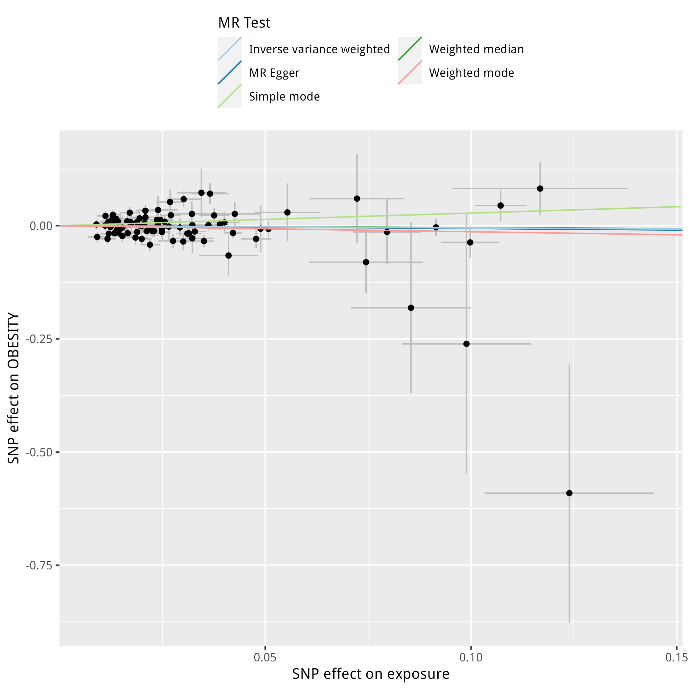

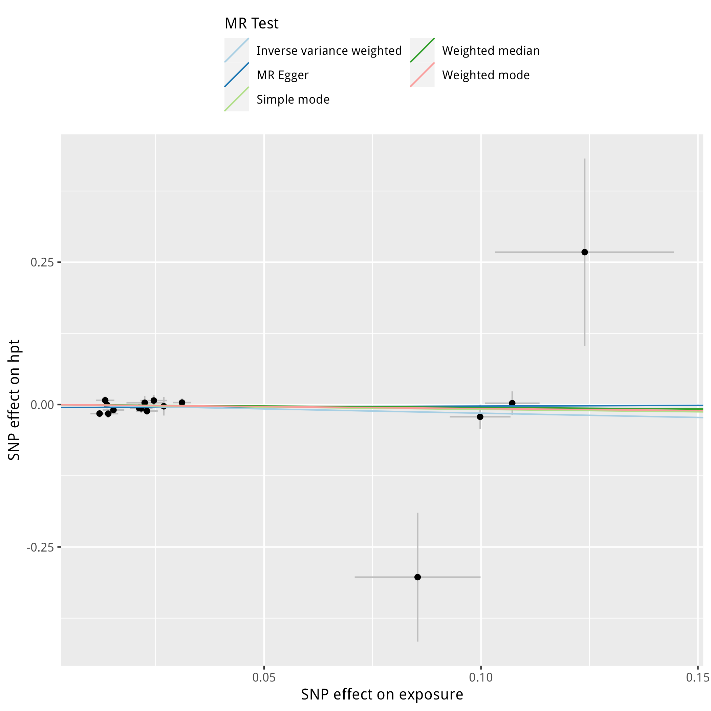


BE

AE

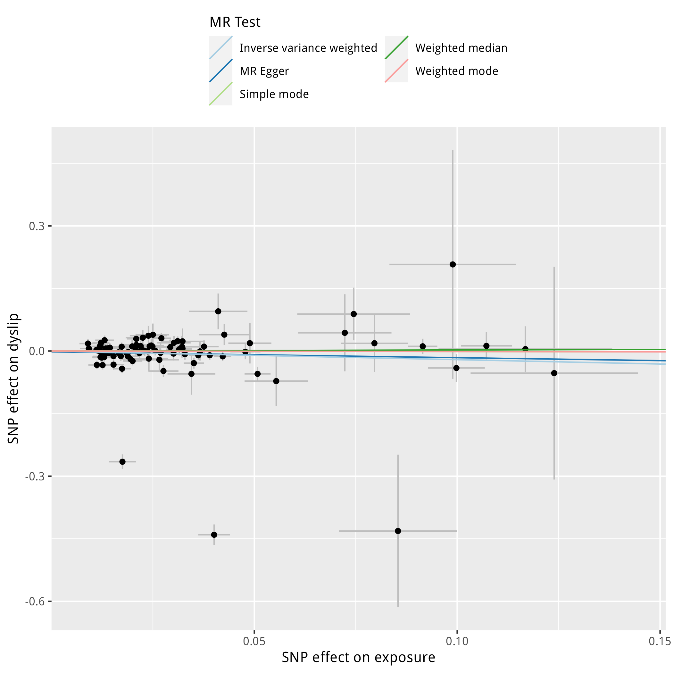

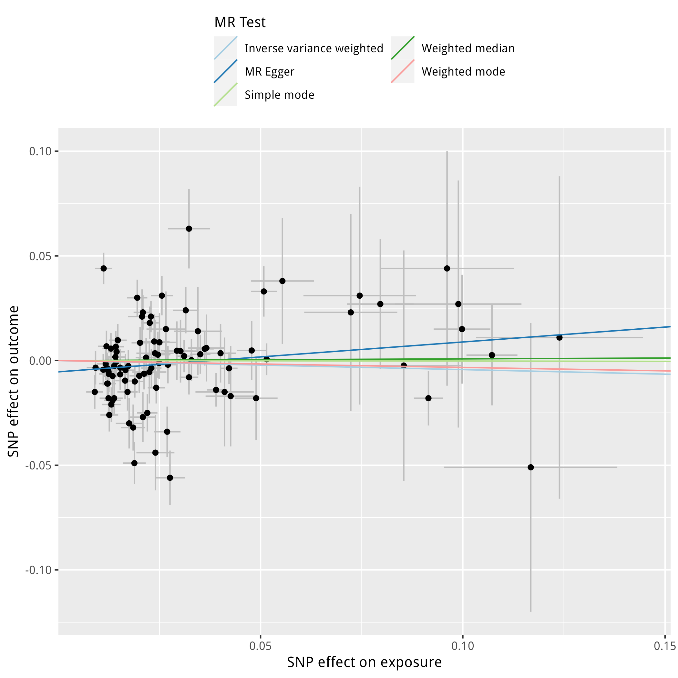


CE

DE

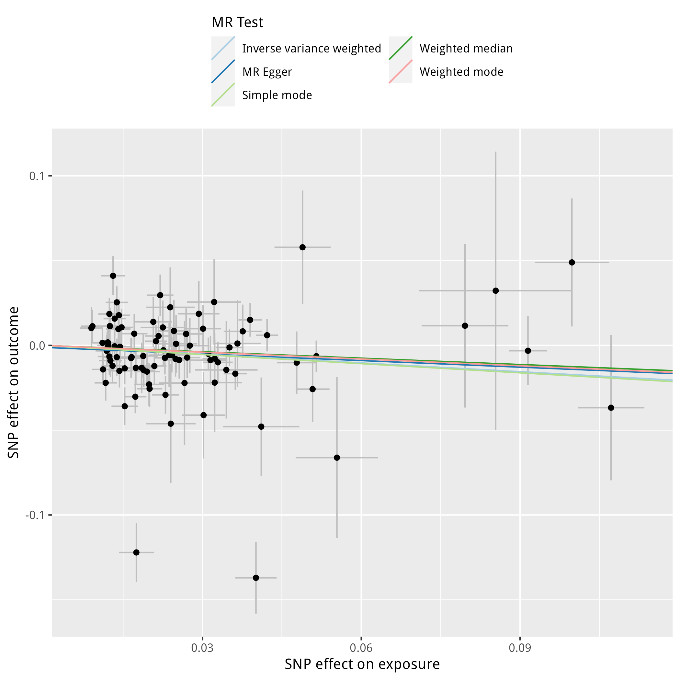

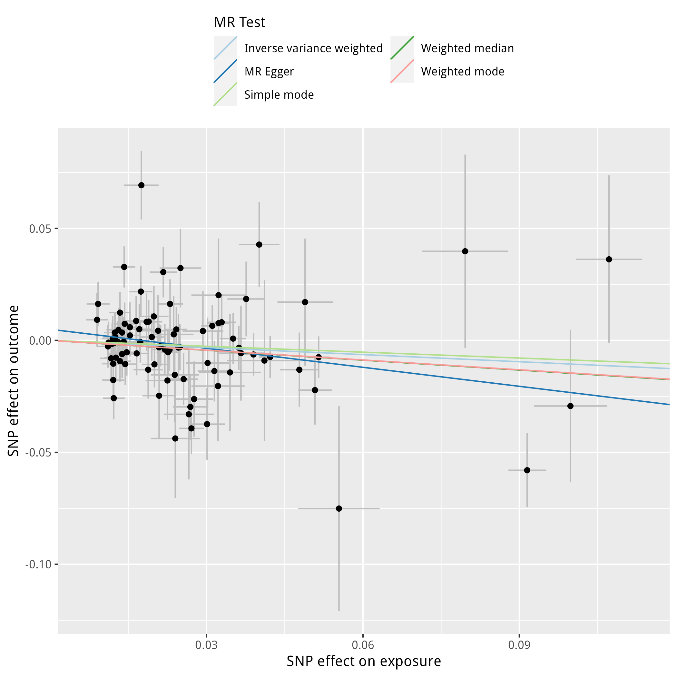


F

E

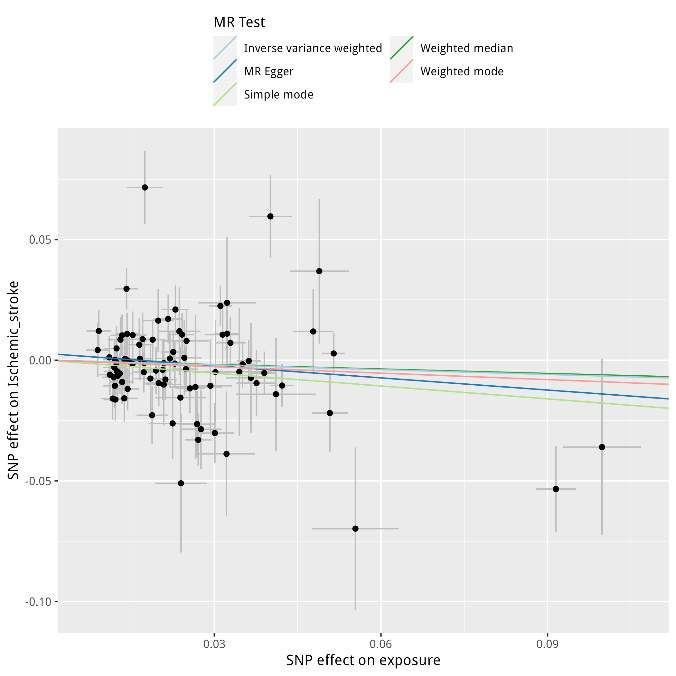

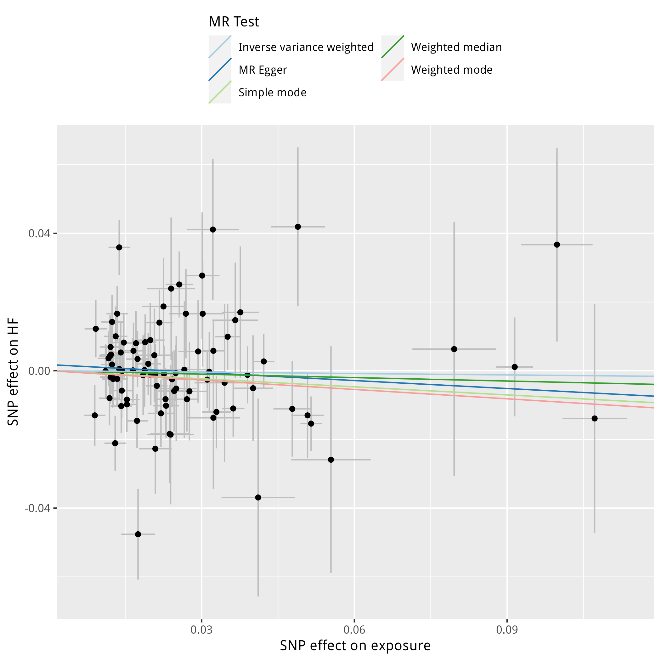


H

G

[**Figure S3**](https://europepmc.org/articles/PMC9349767/figure/jmv28008-fig-0003/) **The forward MR analyses (validation analysis using mtDNA copy number by Longchamps): Scatter plot of the association between mtDNA copy number and cardiometabolic disease.** (A) Obesity, (B) hypertension, (C) dyslipidemia, (D) T2DM, (E) CAD, (F) Stroke, (G) Ischemic stroke, (H) Heart failure. The four methods applied in the current manuscript were all depicted. Lines in black, red, green, and blue represent IVW, MR‐Egger, weighted median, and weight mode methods.


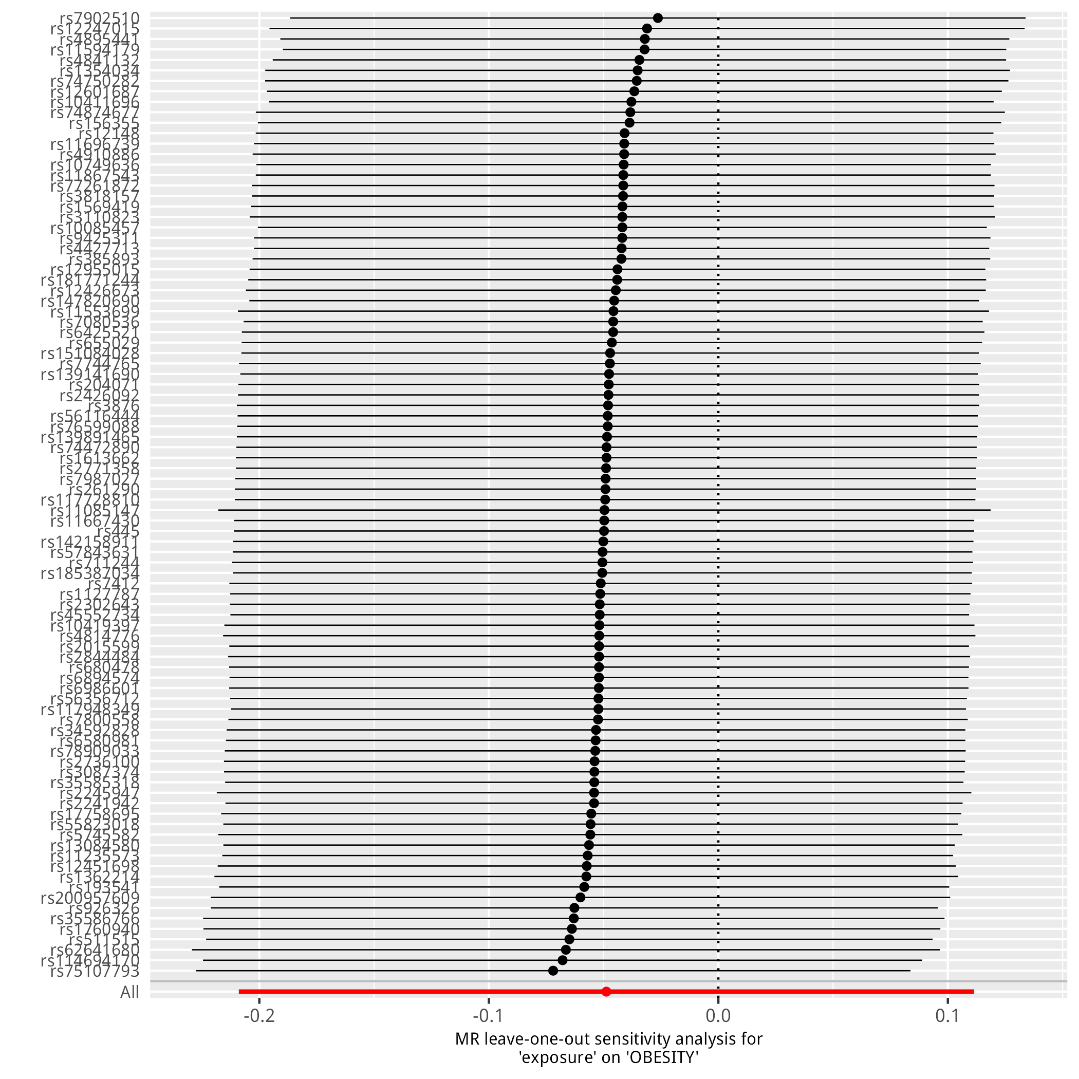

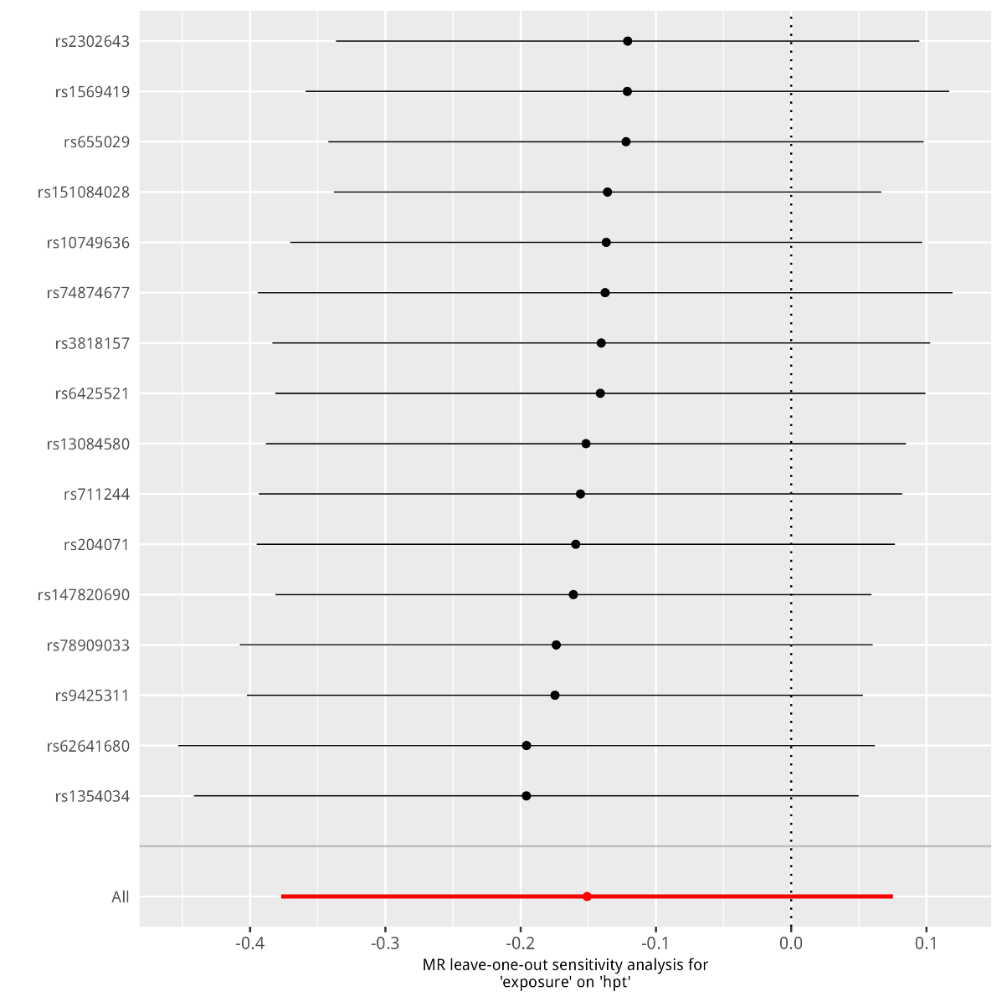


B


A

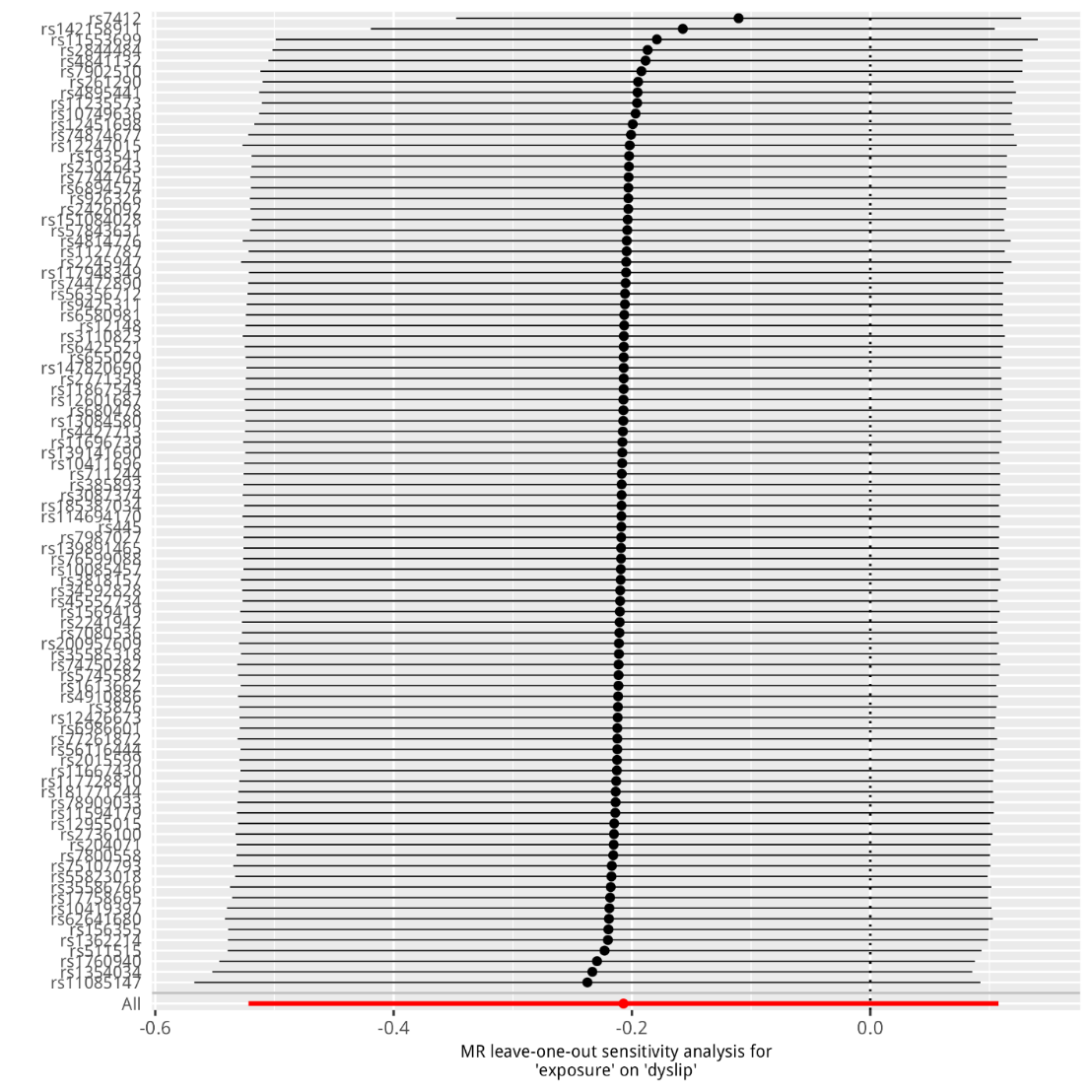

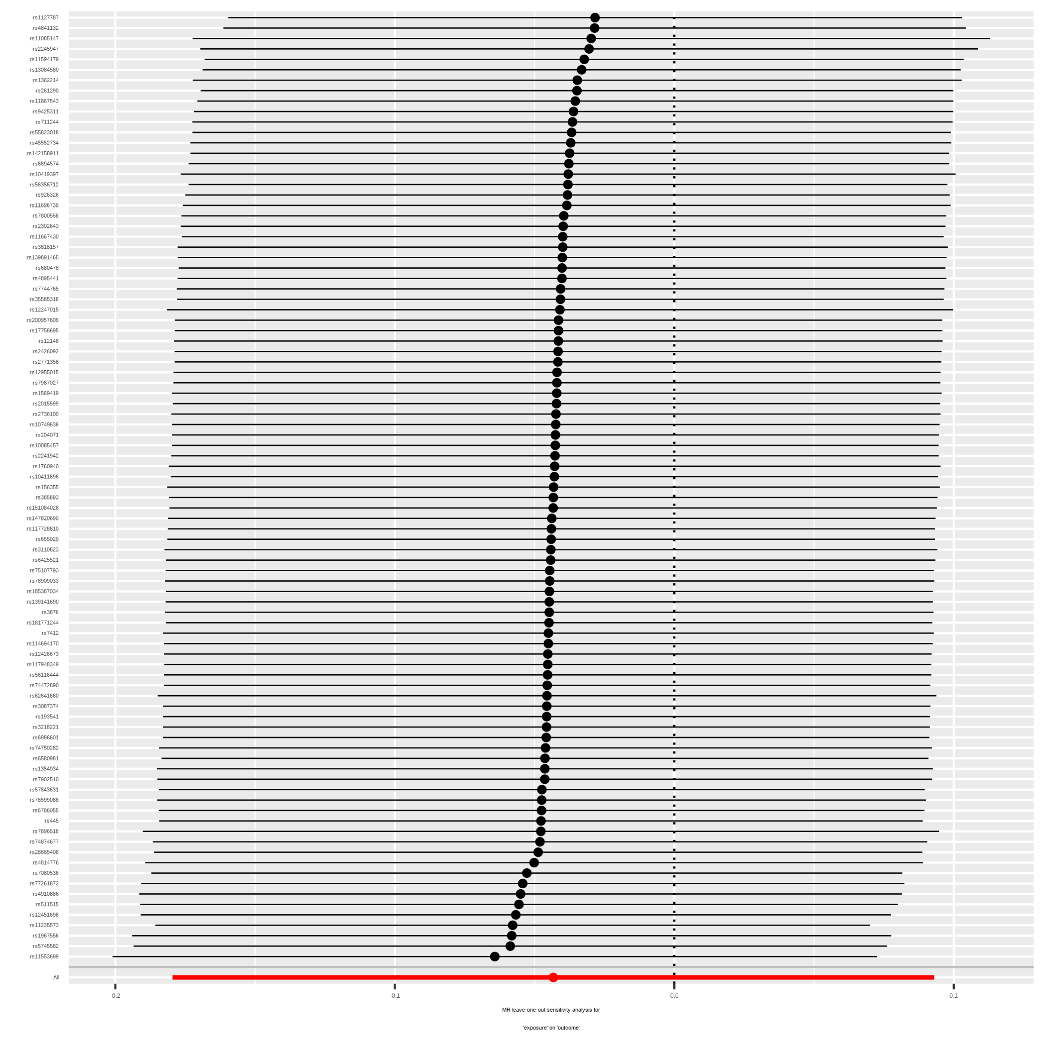


D

C

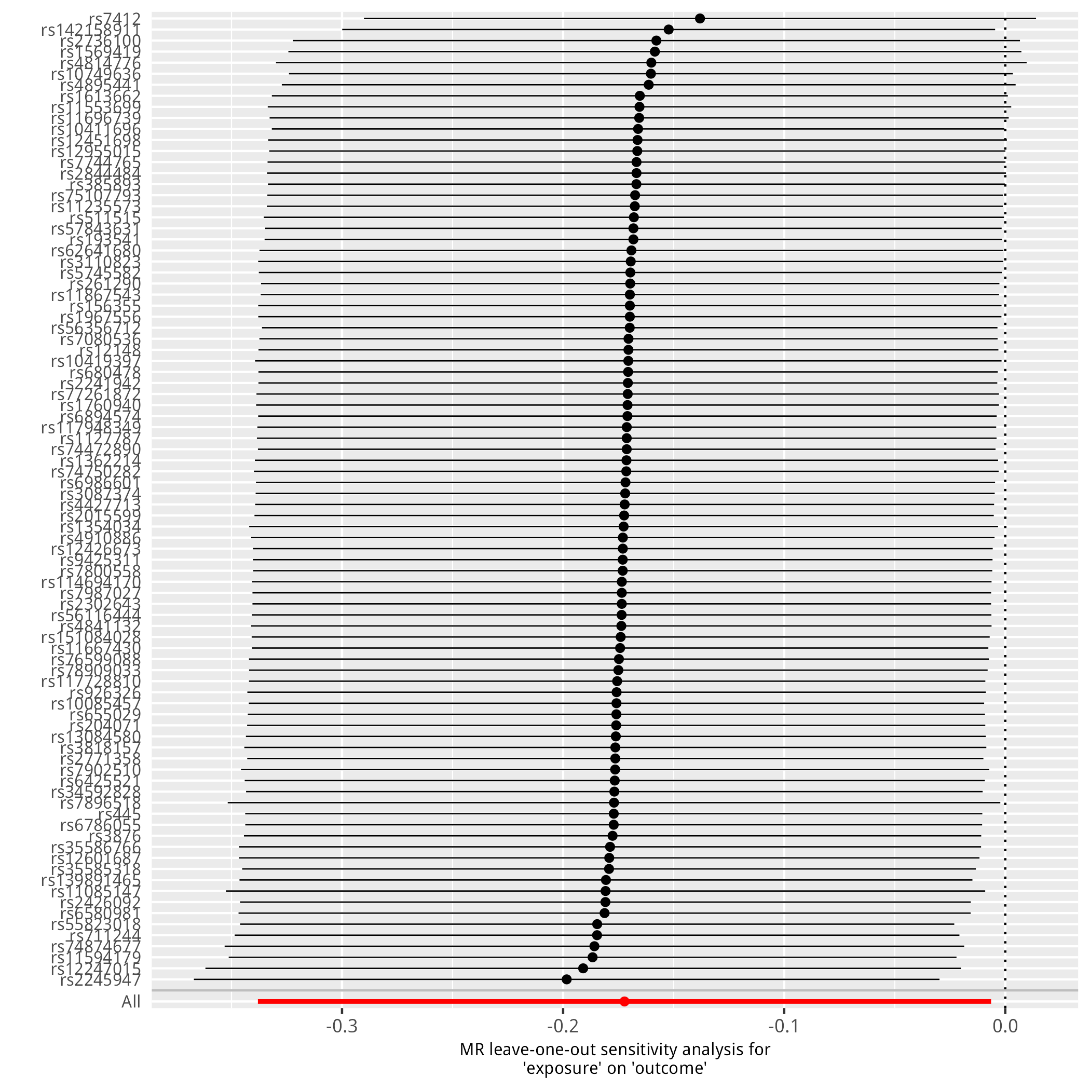

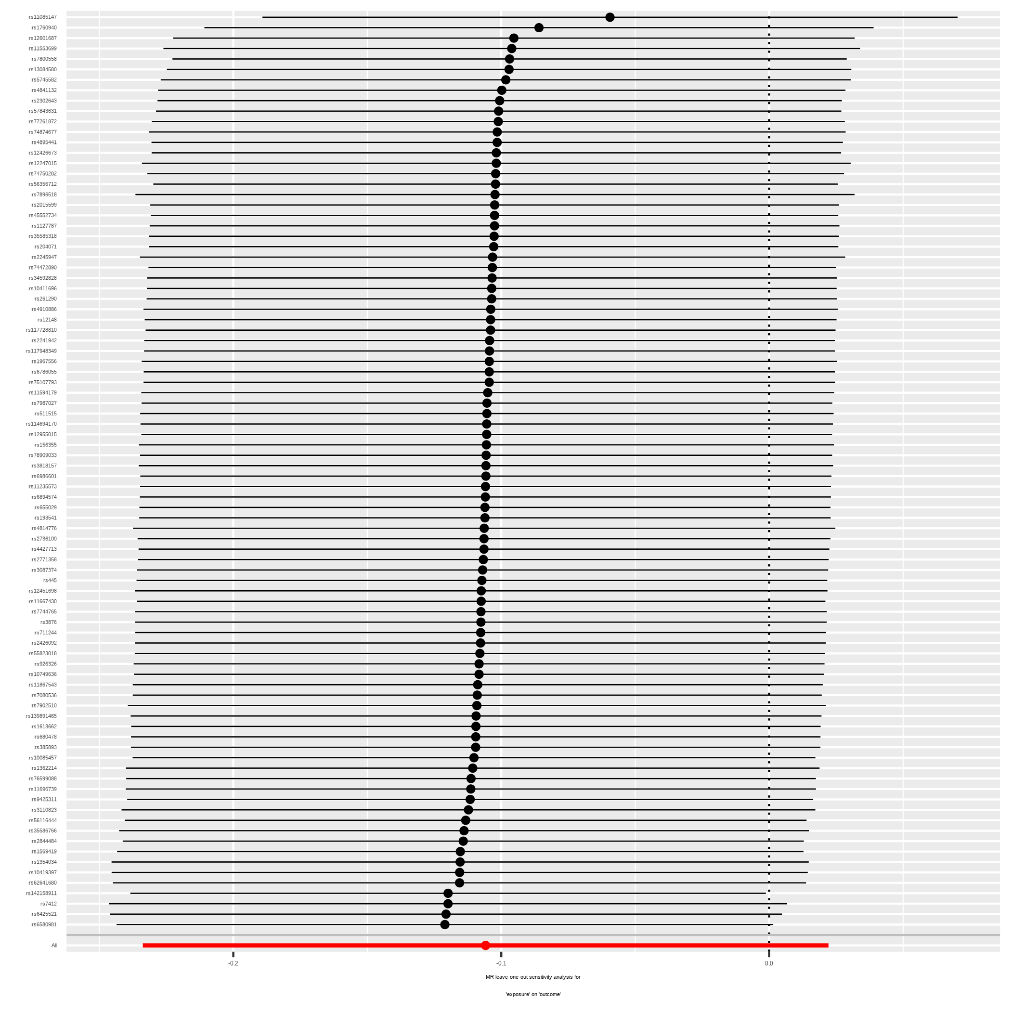

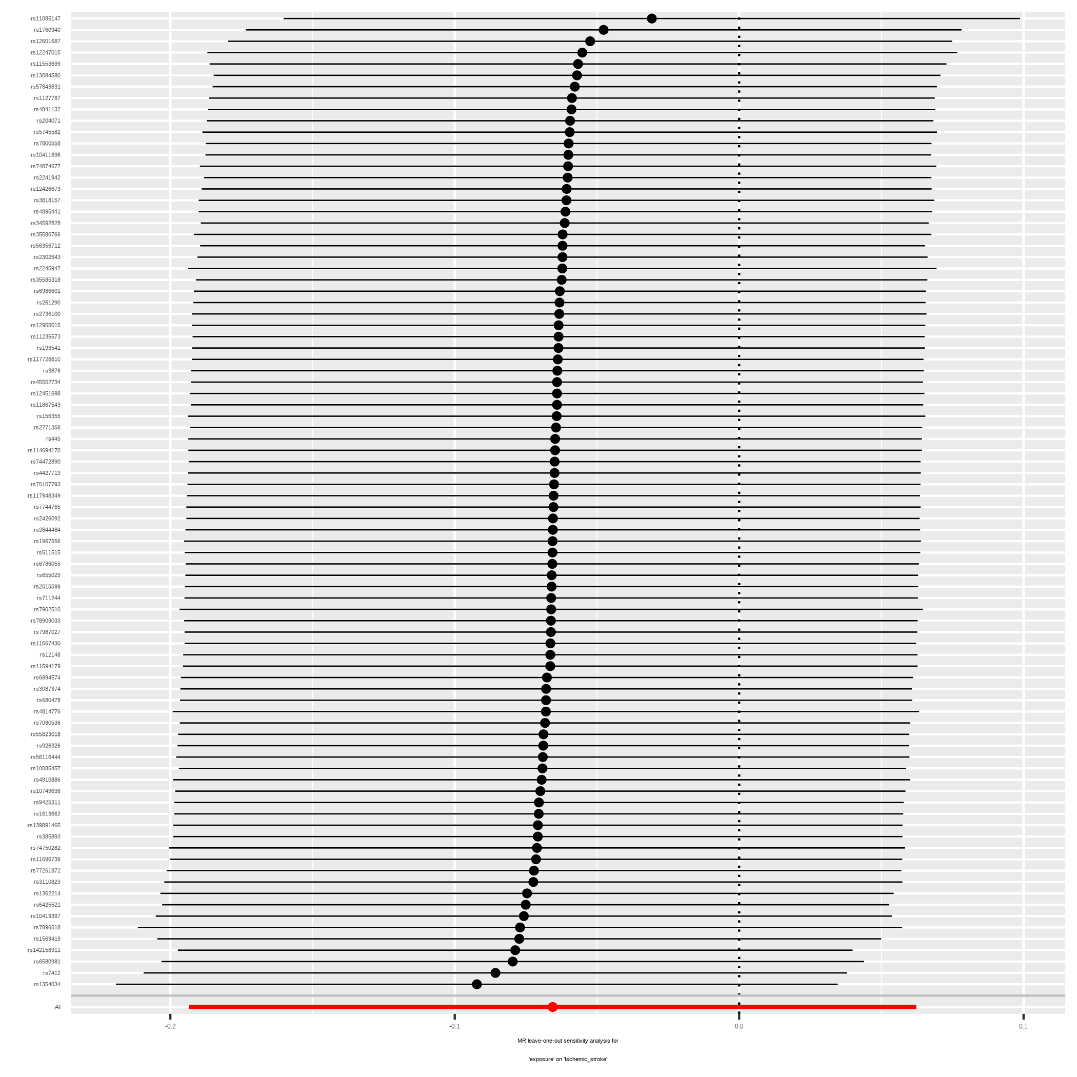

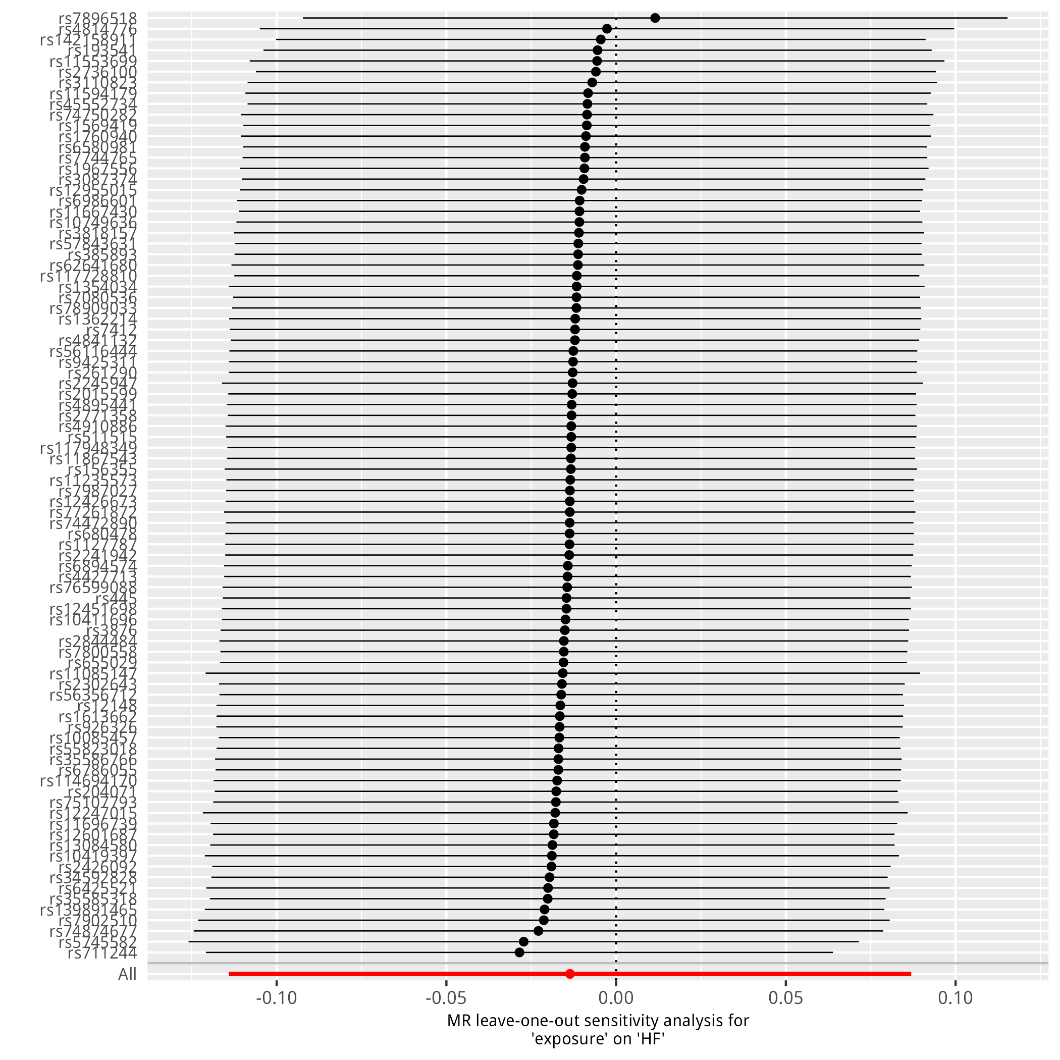


F

E


**Figure S4 The forward MR analyses (validation analysis using mtDNA copy number by Longchamps). Plots of “leave-one-out” analyses for MR analyses.** (A) Obesity, (B) hypertension, (C) dyslipidemia, (D) T2DM, (E) CAD, (F) Stroke, (G) Ischemic stroke, (H) Heart failure. The horizontal lines in the figure represents beta value and its 95% confidence interval [CI] of causal inference, which indicates the genetic effect of the SNP on cardiometabolic disease.

H

G
